# Supplementary figures and images for: CRISPR-Cas12a exhibits metal-dependent specificity switching
Source: bioRxiv. 2024 Jan 17:2023.11.29.569287. Originally published 2023 Nov 29. Preprint. [Version 2] doi: 10.1101/2023.11.29.569287 (PMC10705449; doi:10.1101/2023.11.29.569287)

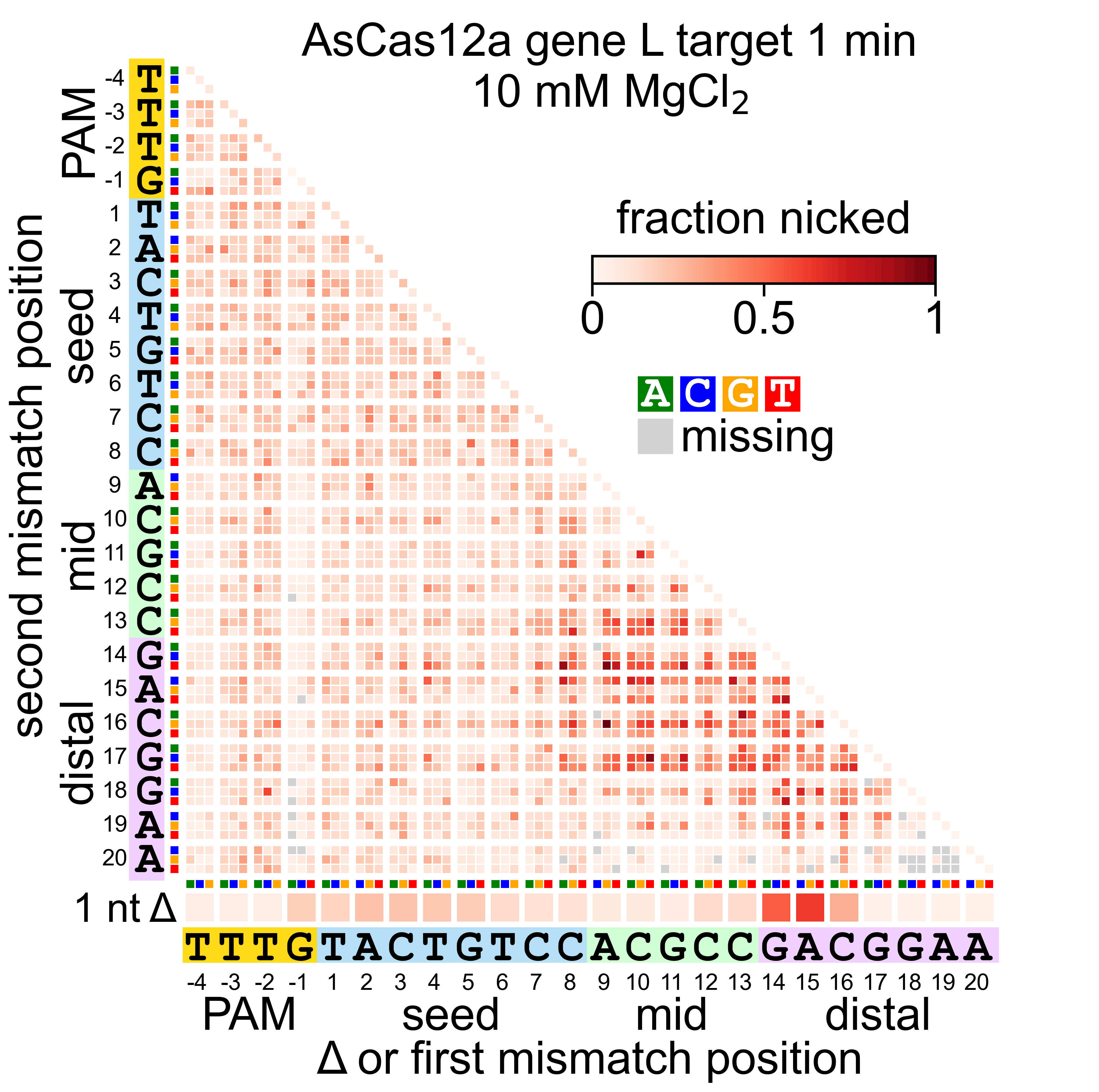

Supplement: Supplement 1 [file media-1.zip › Supplementary_Data_1/fraction_nicked_gifs/As_L_1_nicked.gif]

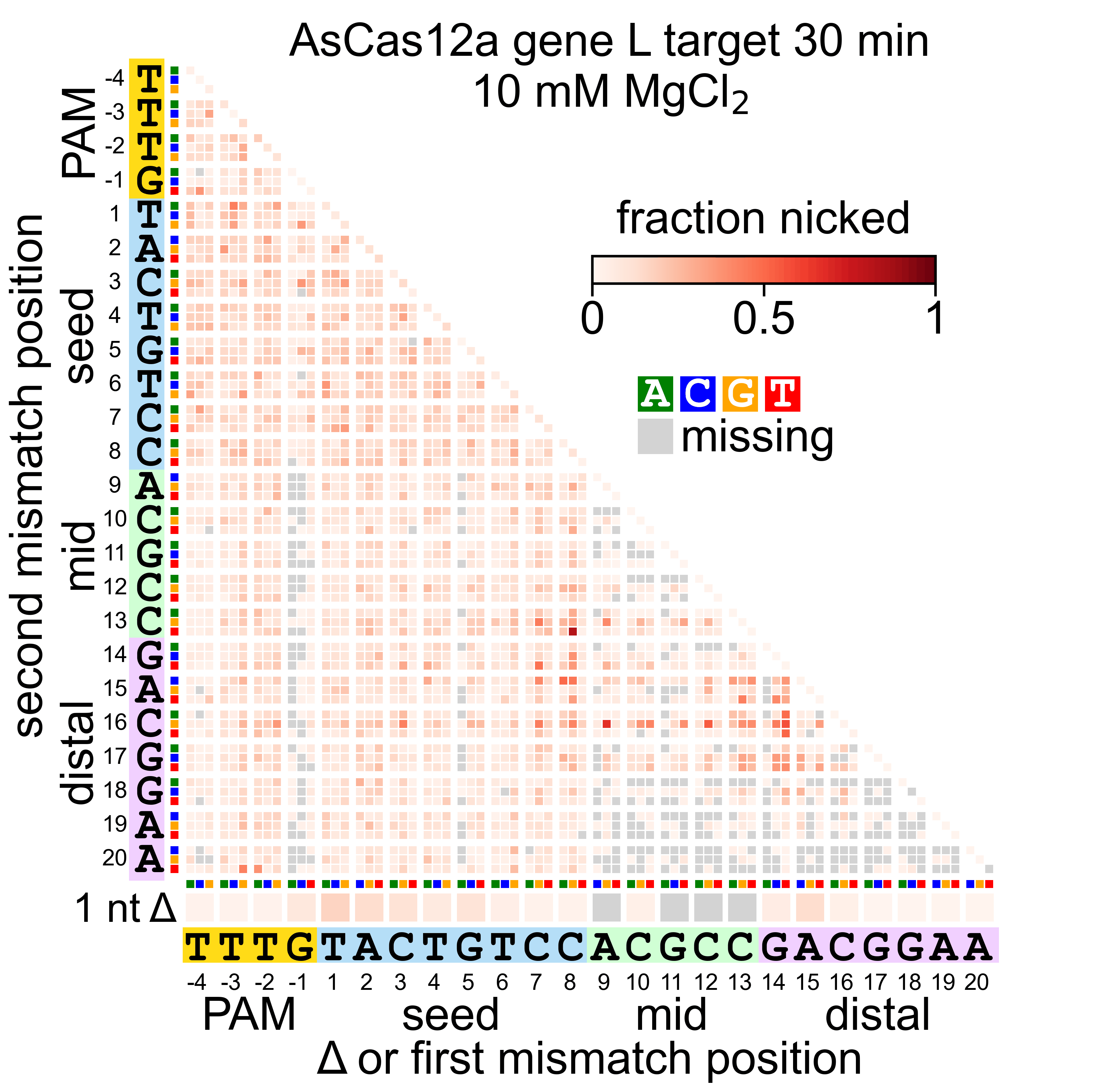

Supplement: Supplement 1 [file media-1.zip › Supplementary_Data_1/fraction_nicked_gifs/As_L_30_nicked.gif]

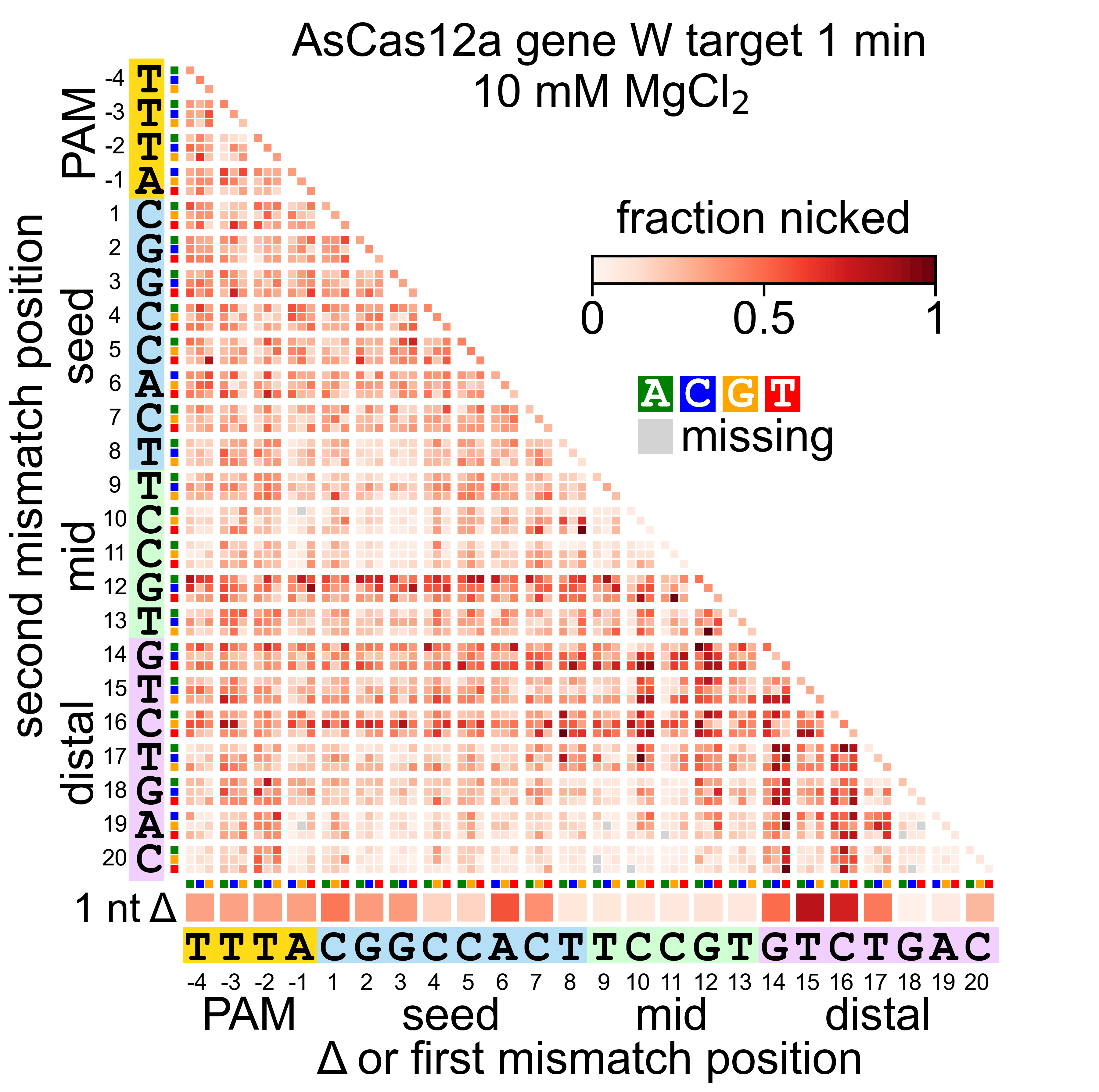

Supplement: Supplement 1 [file media-1.zip › Supplementary_Data_1/fraction_nicked_gifs/As_W_1_nicked.gif]

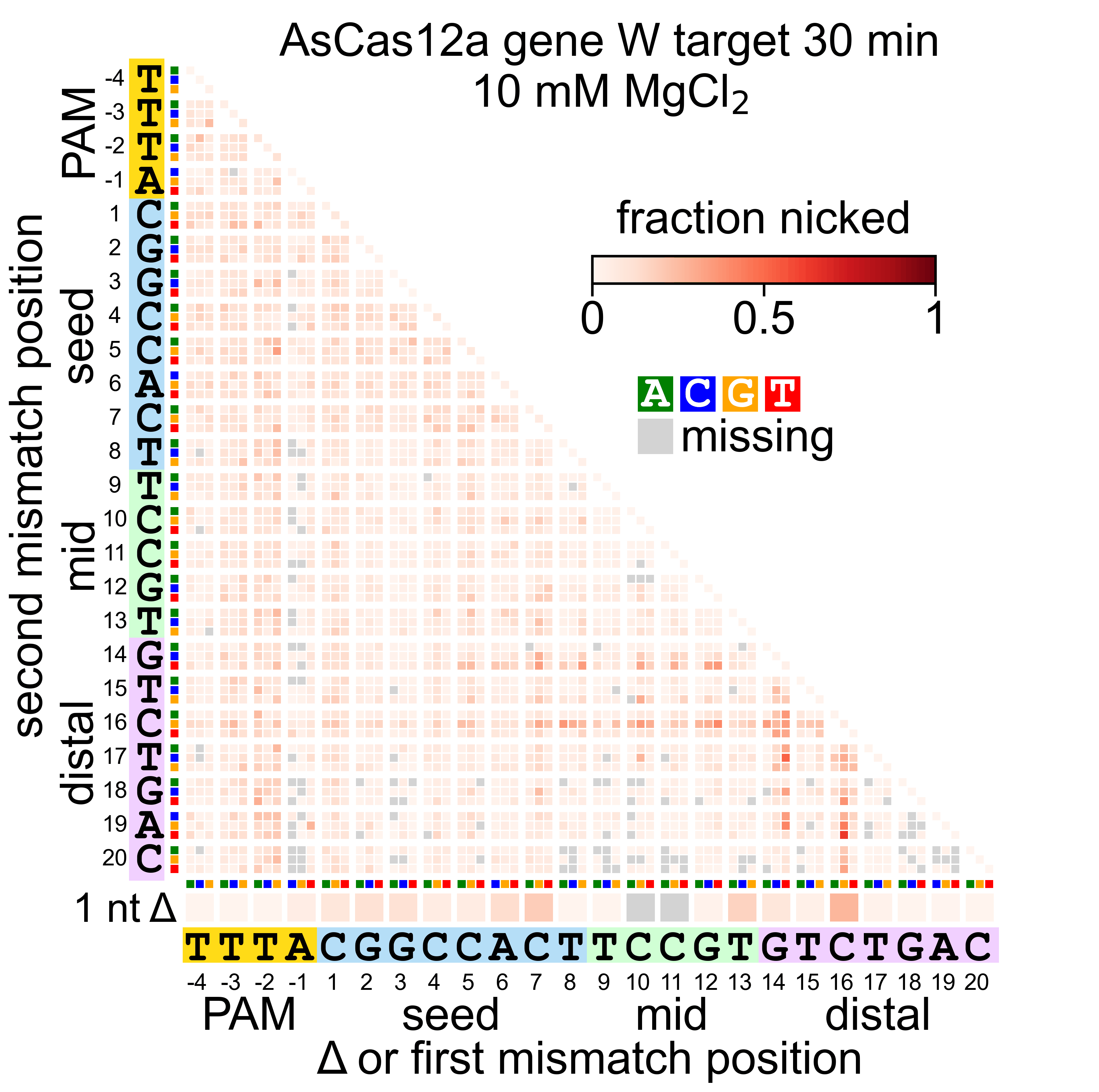

Supplement: Supplement 1 [file media-1.zip › Supplementary_Data_1/fraction_nicked_gifs/As_W_30_nicked.gif]

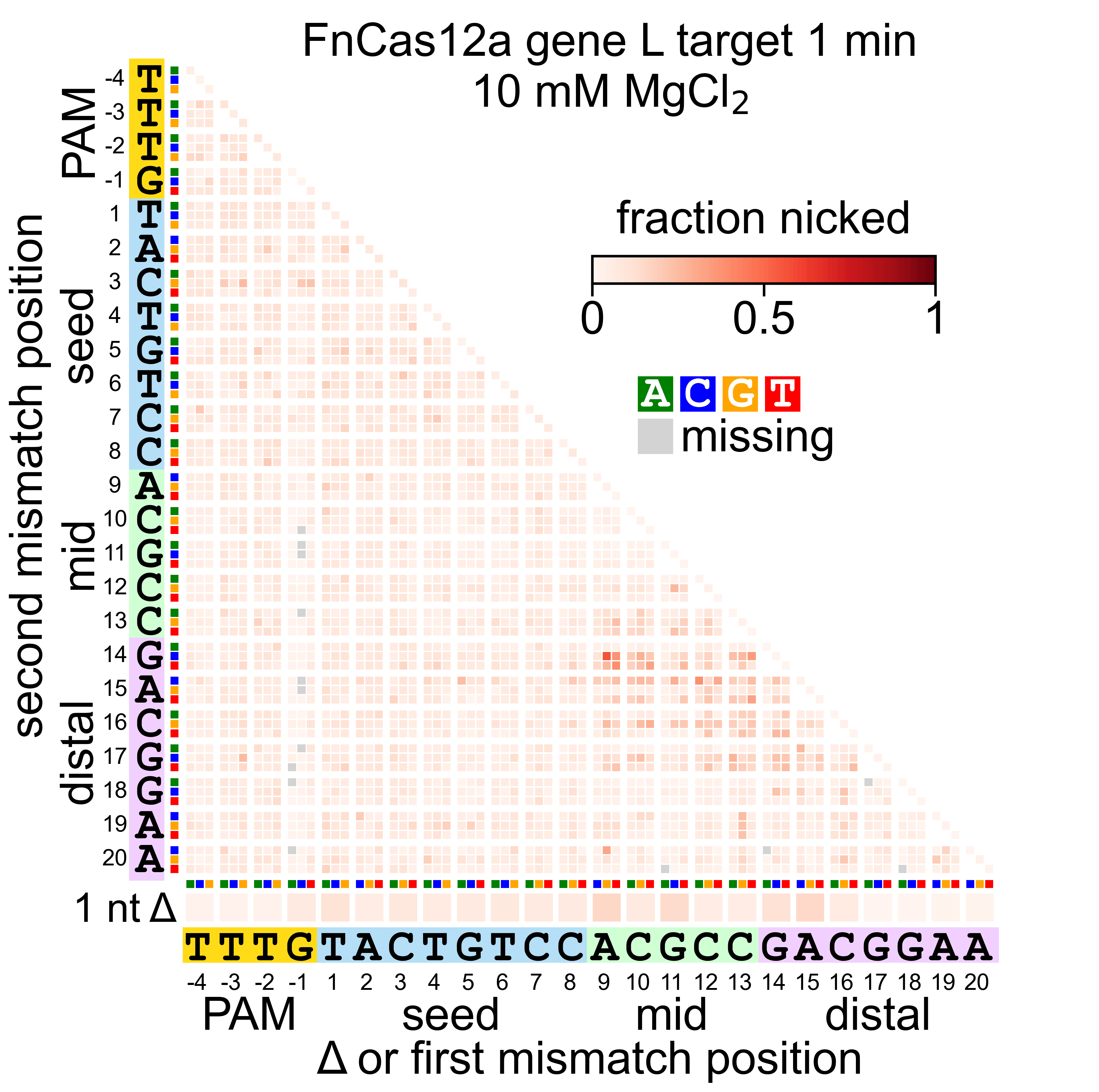

Supplement: Supplement 1 [file media-1.zip › Supplementary_Data_1/fraction_nicked_gifs/Fn_L_1_nicked.gif]

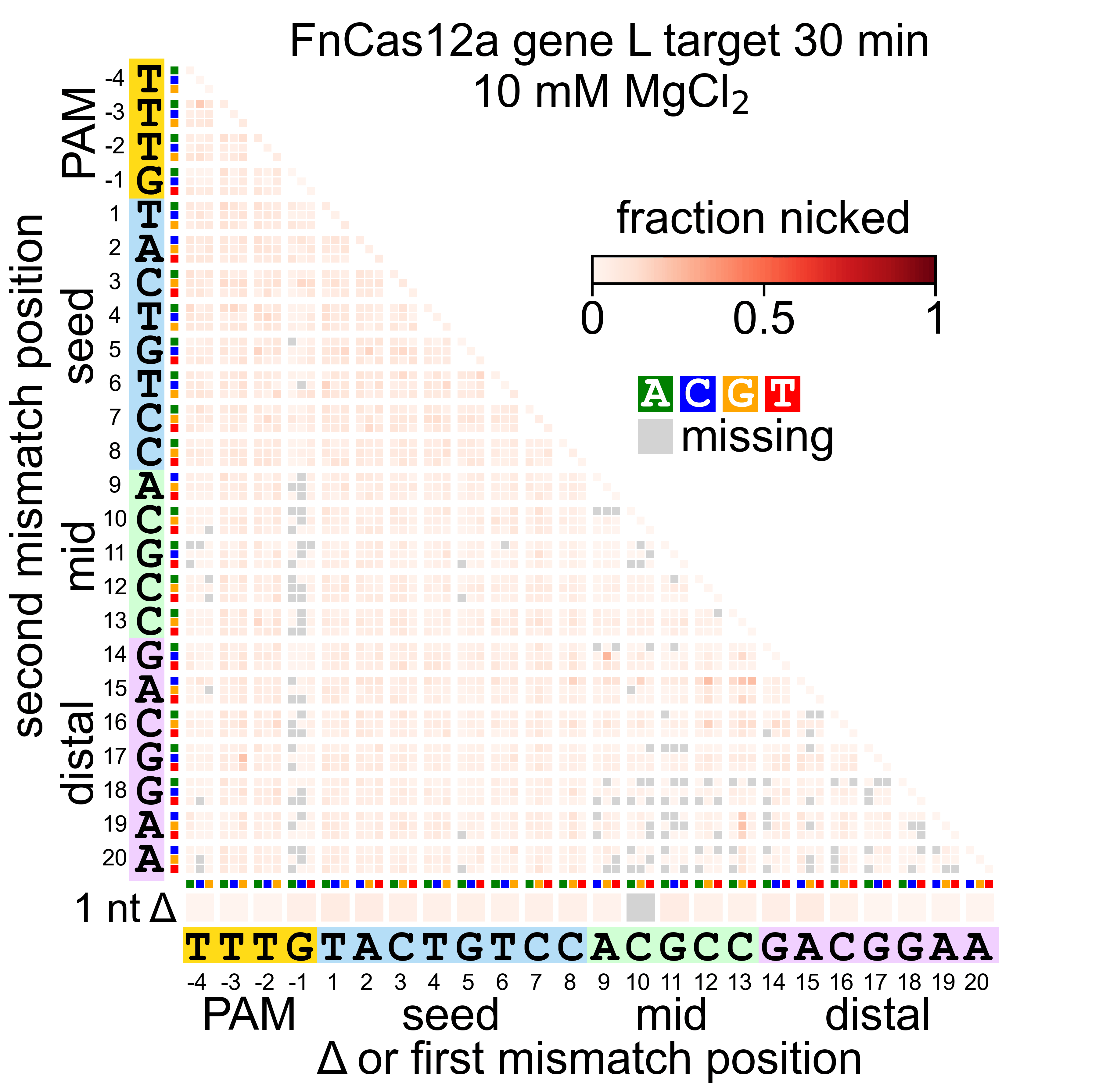

Supplement: Supplement 1 [file media-1.zip › Supplementary_Data_1/fraction_nicked_gifs/Fn_L_30_nicked.gif]

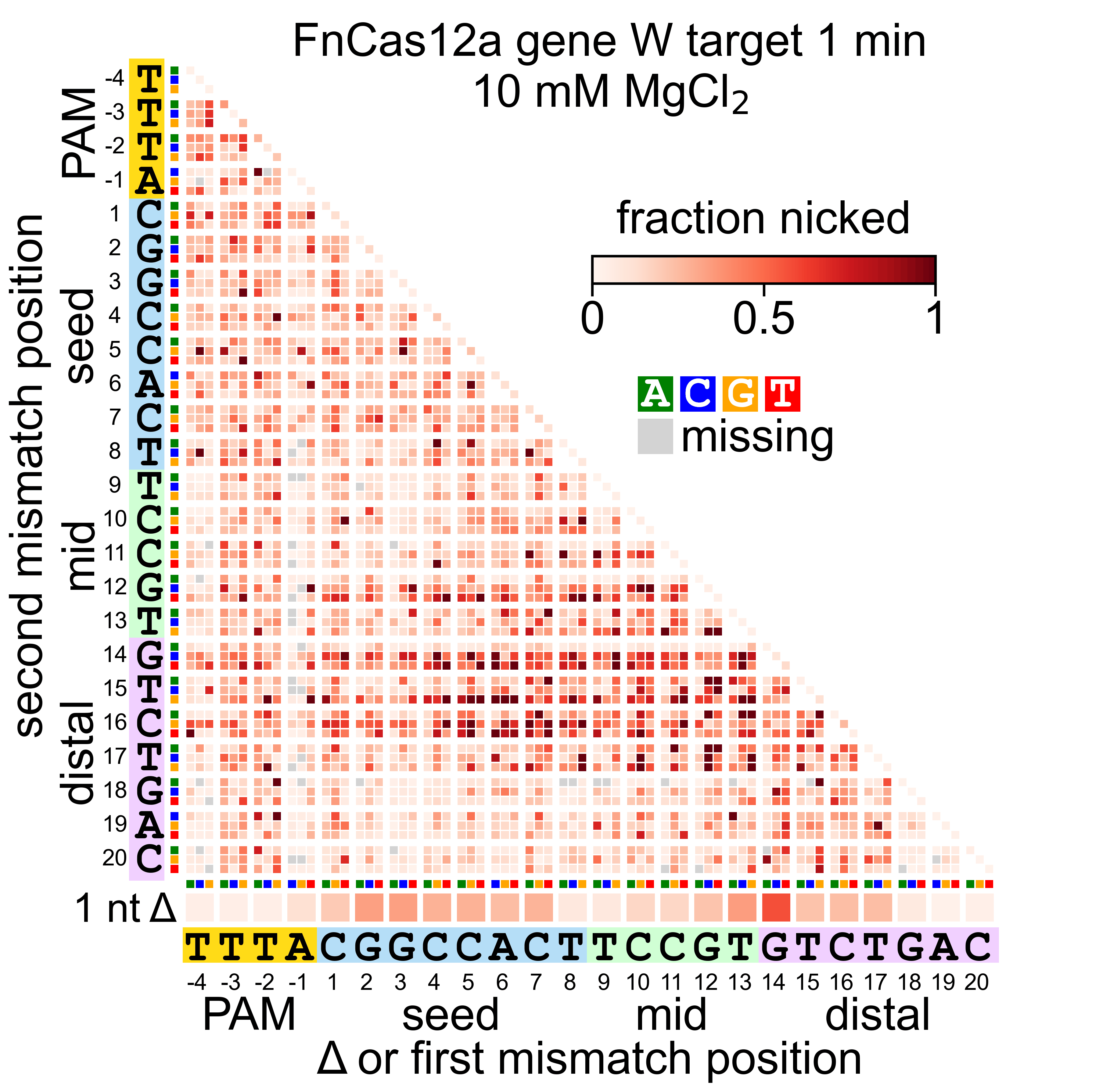

Supplement: Supplement 1 [file media-1.zip › Supplementary_Data_1/fraction_nicked_gifs/Fn_W_1_nicked.gif]

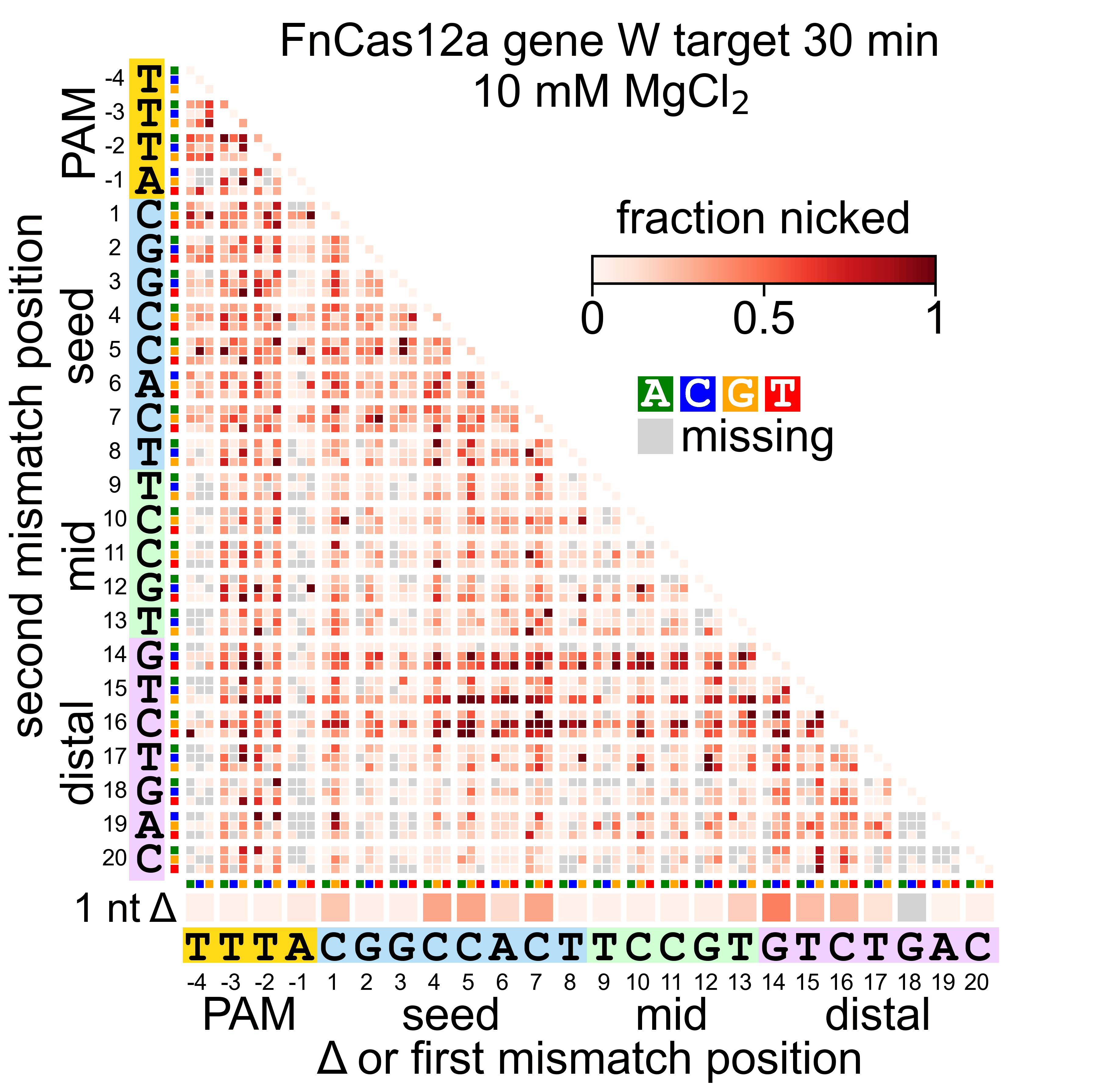

Supplement: Supplement 1 [file media-1.zip › Supplementary_Data_1/fraction_nicked_gifs/Fn_W_30_nicked.gif]

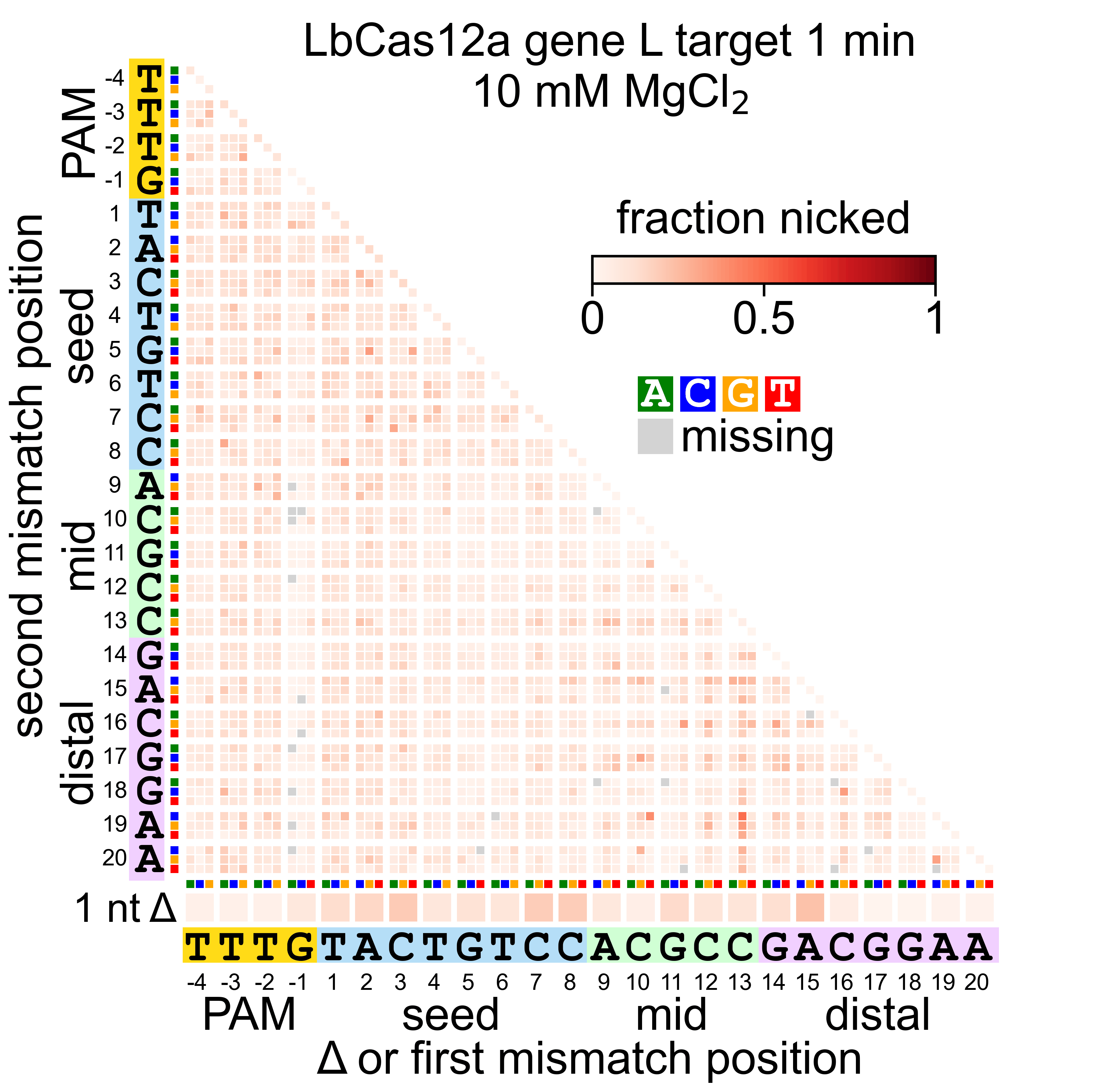

Supplement: Supplement 1 [file media-1.zip › Supplementary_Data_1/fraction_nicked_gifs/Lb_L_1_nicked.gif]

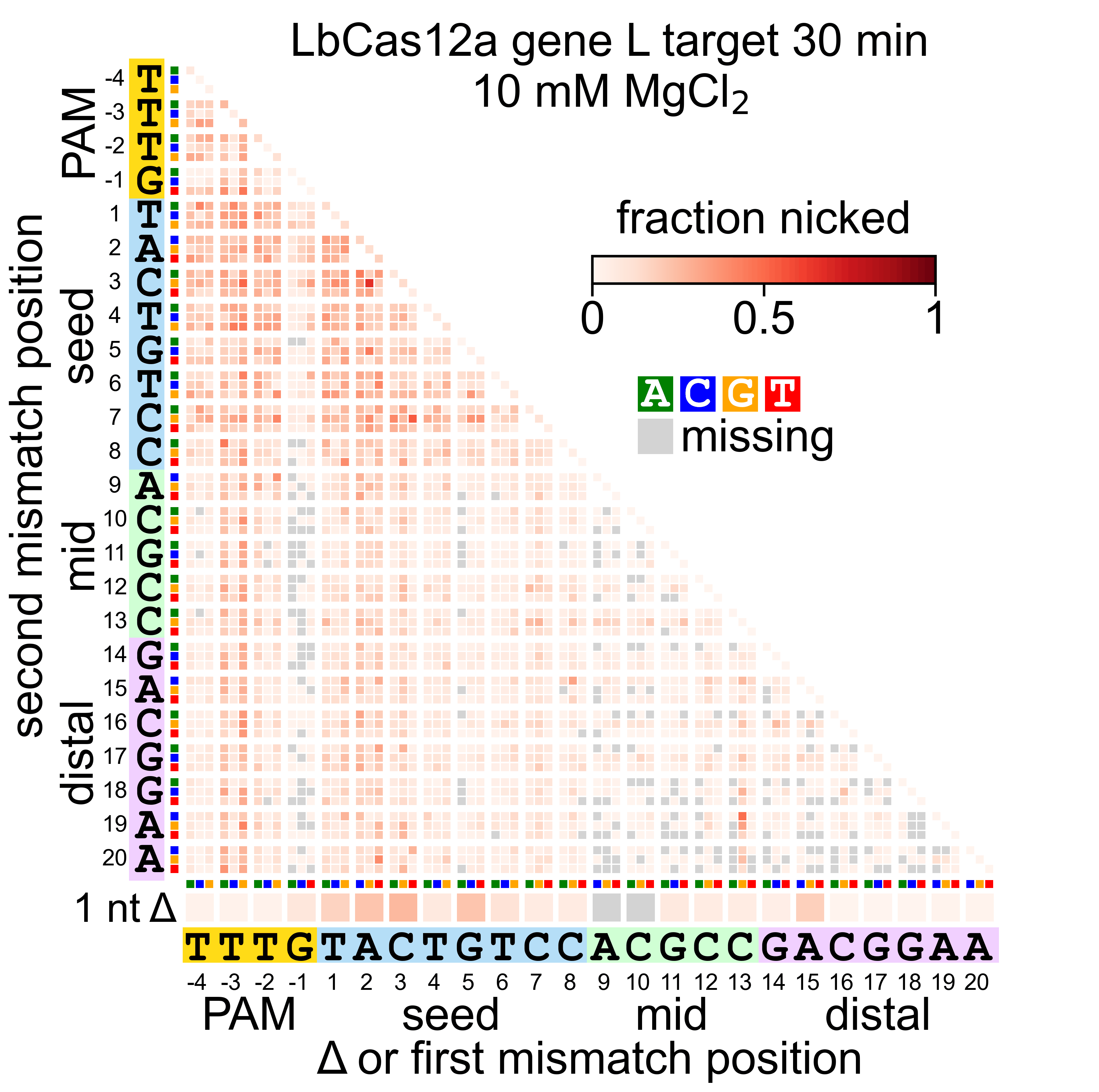

Supplement: Supplement 1 [file media-1.zip › Supplementary_Data_1/fraction_nicked_gifs/Lb_L_30_nicked.gif]

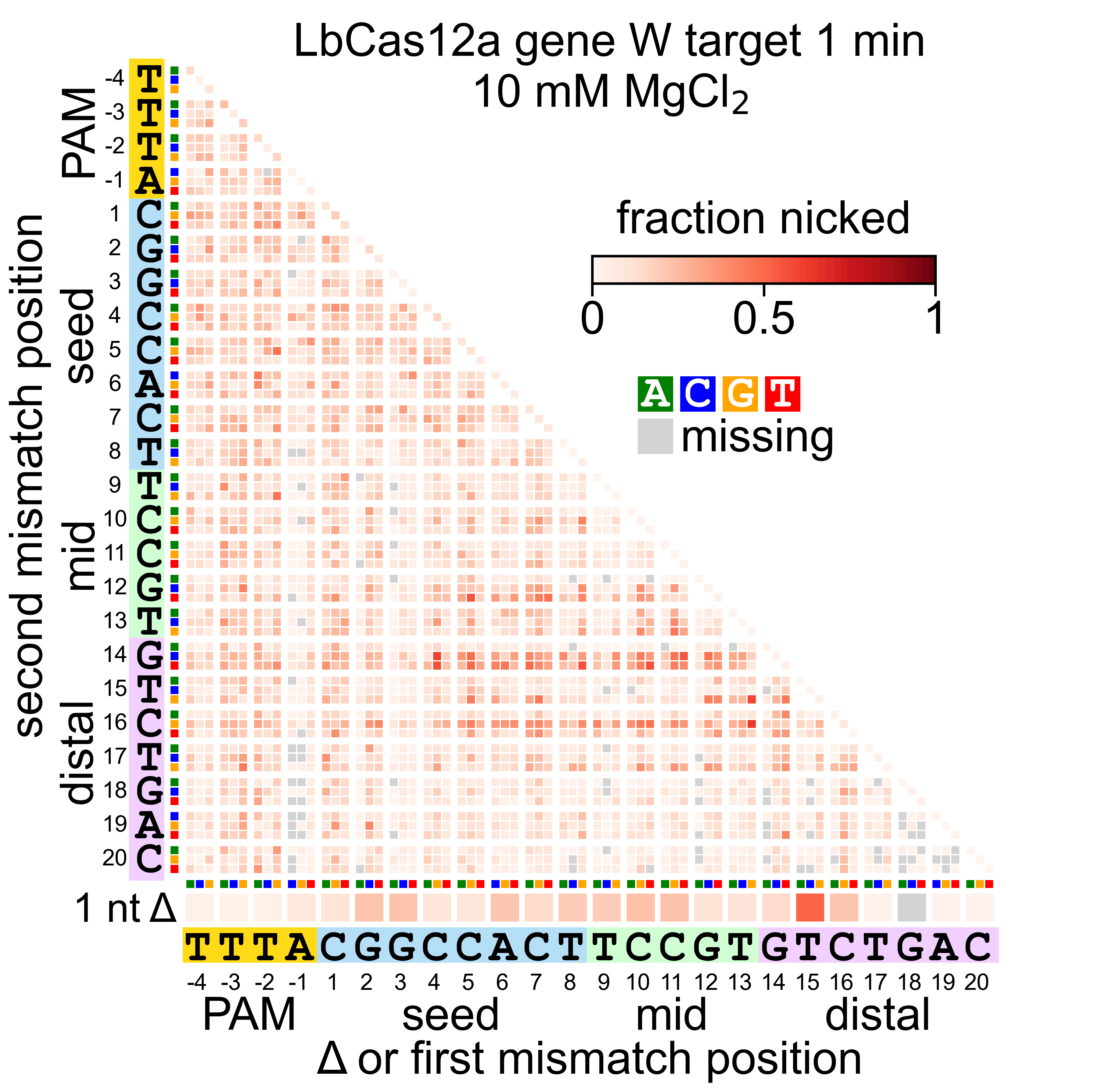

Supplement: Supplement 1 [file media-1.zip › Supplementary_Data_1/fraction_nicked_gifs/Lb_W_1_nicked.gif]

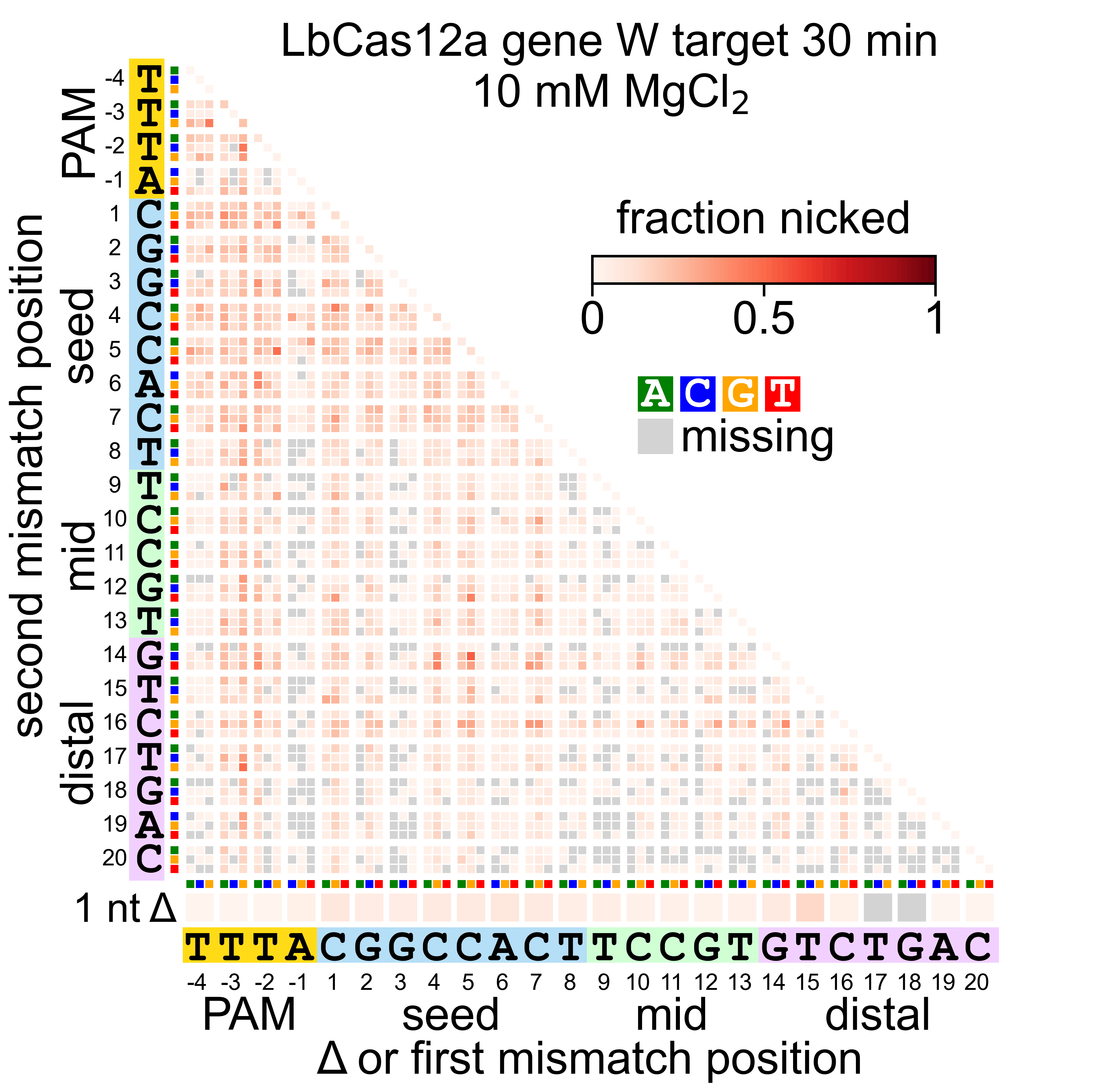

Supplement: Supplement 1 [file media-1.zip › Supplementary_Data_1/fraction_nicked_gifs/Lb_W_30_nicked.gif]

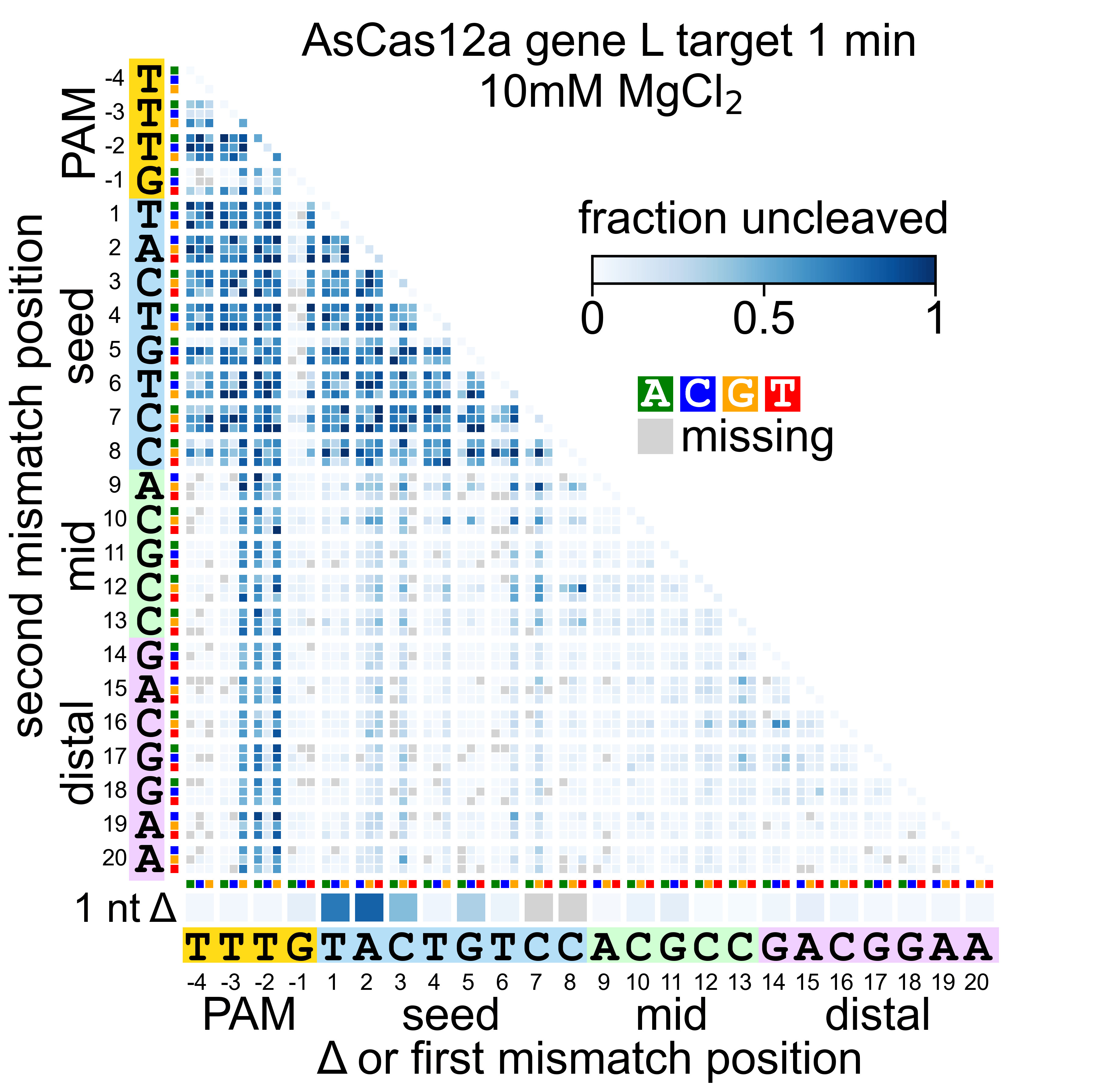

Supplement: Supplement 1 [file media-1.zip › Supplementary_Data_1/fraction_uncleaved_gifs/As_L_1_uncleaved.gif]

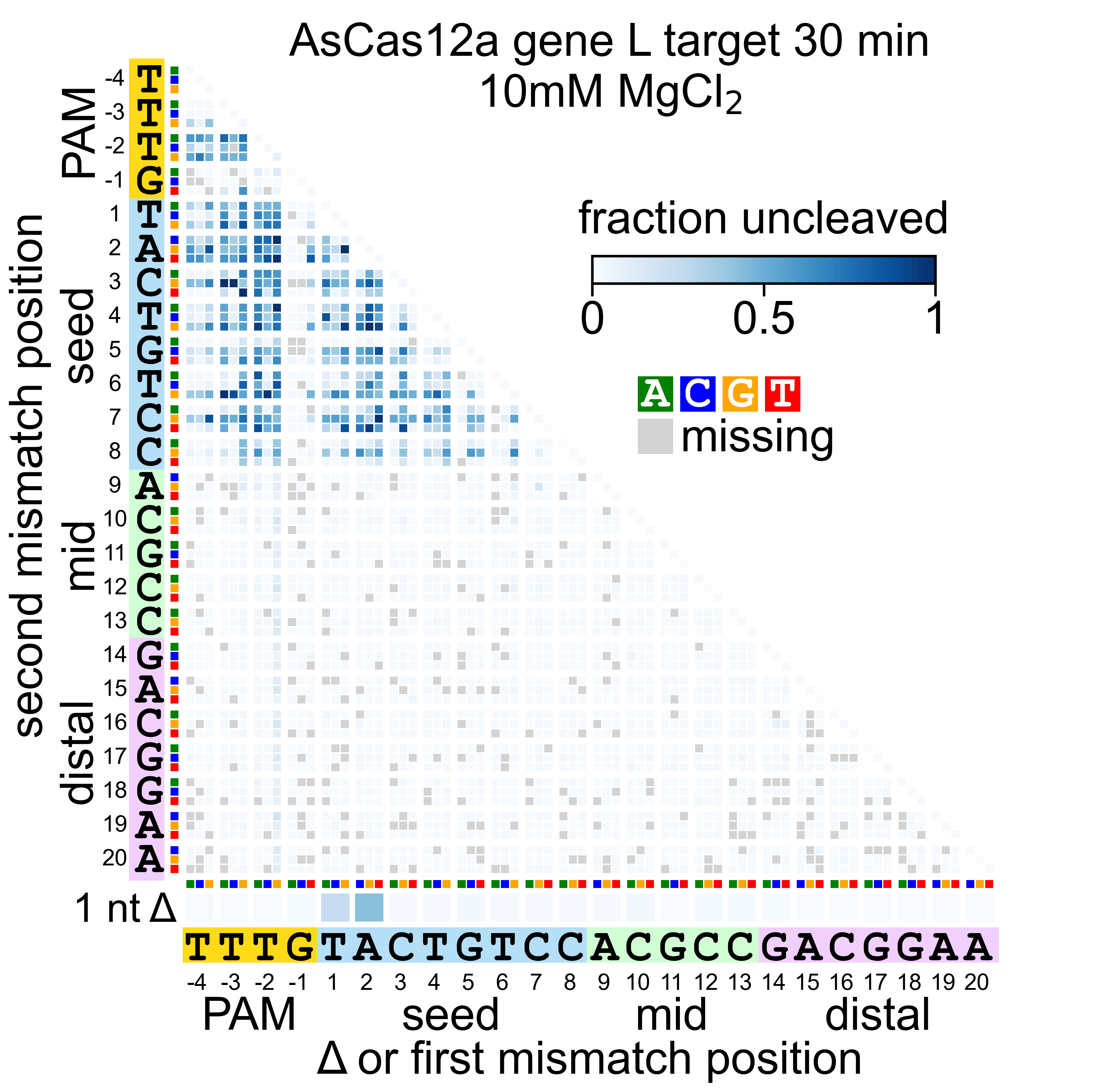

Supplement: Supplement 1 [file media-1.zip › Supplementary_Data_1/fraction_uncleaved_gifs/As_L_30_uncleaved.gif]

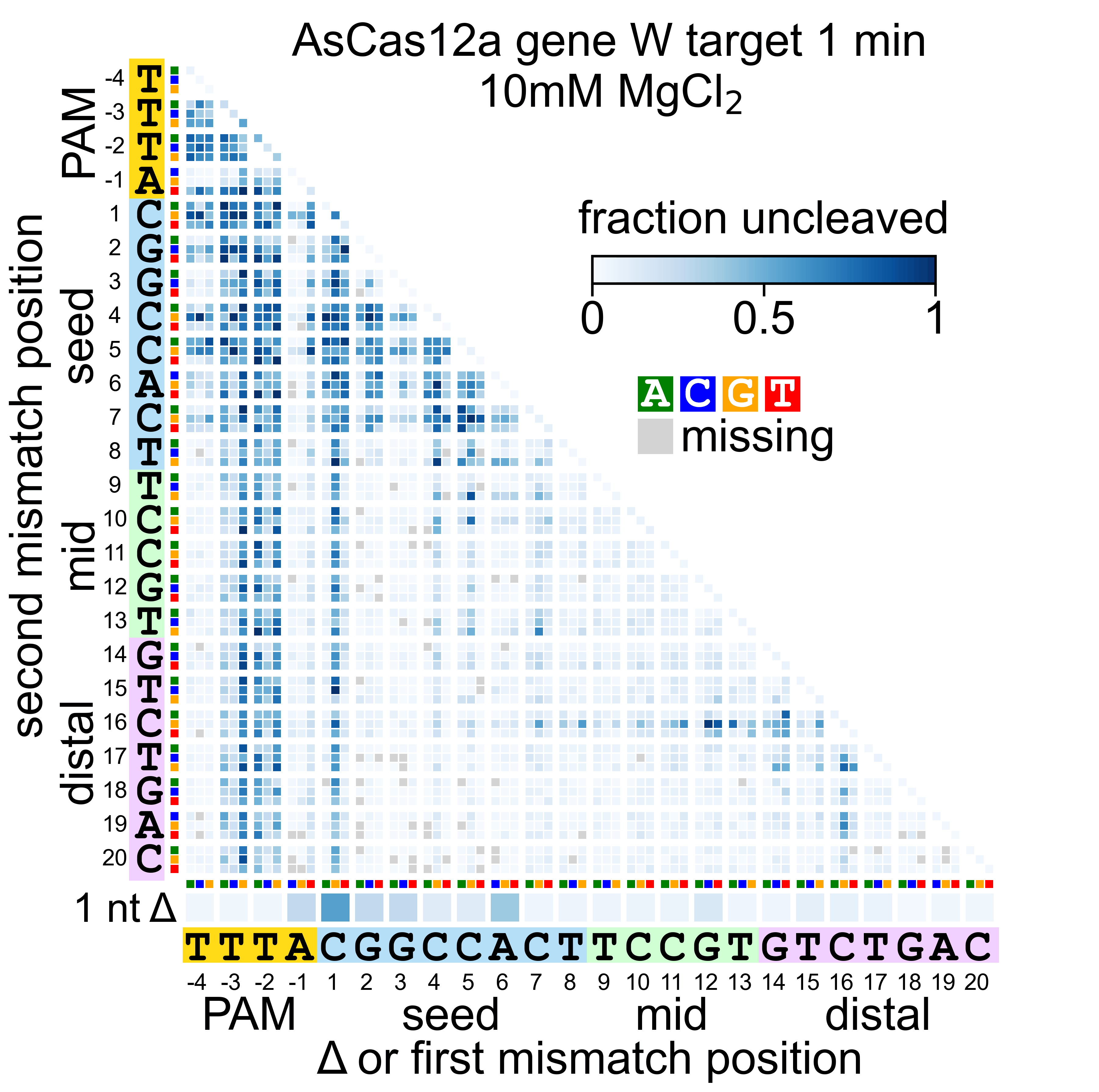

Supplement: Supplement 1 [file media-1.zip › Supplementary_Data_1/fraction_uncleaved_gifs/As_W_1_uncleaved.gif]

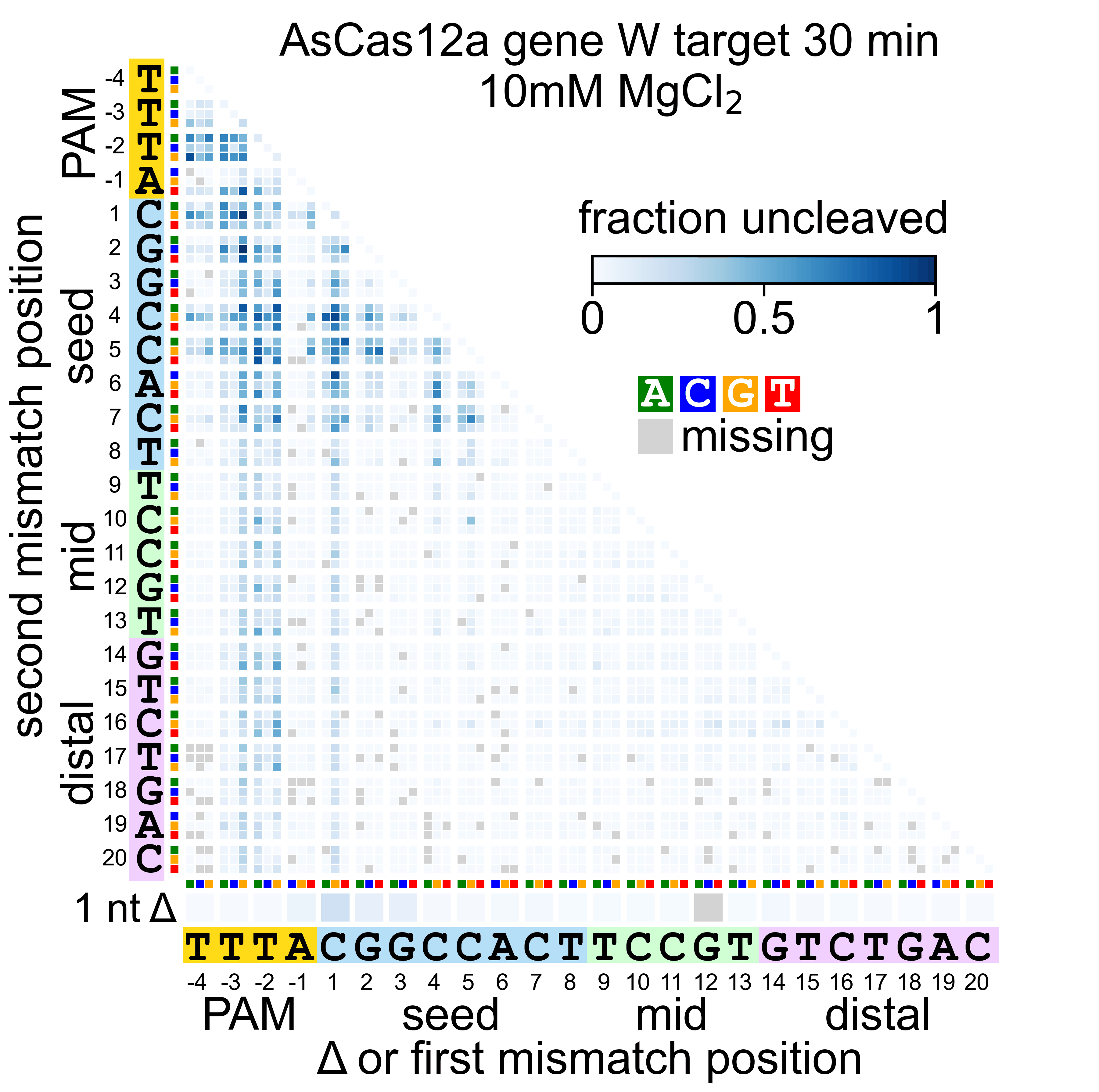

Supplement: Supplement 1 [file media-1.zip › Supplementary_Data_1/fraction_uncleaved_gifs/As_W_30_uncleaved.gif]

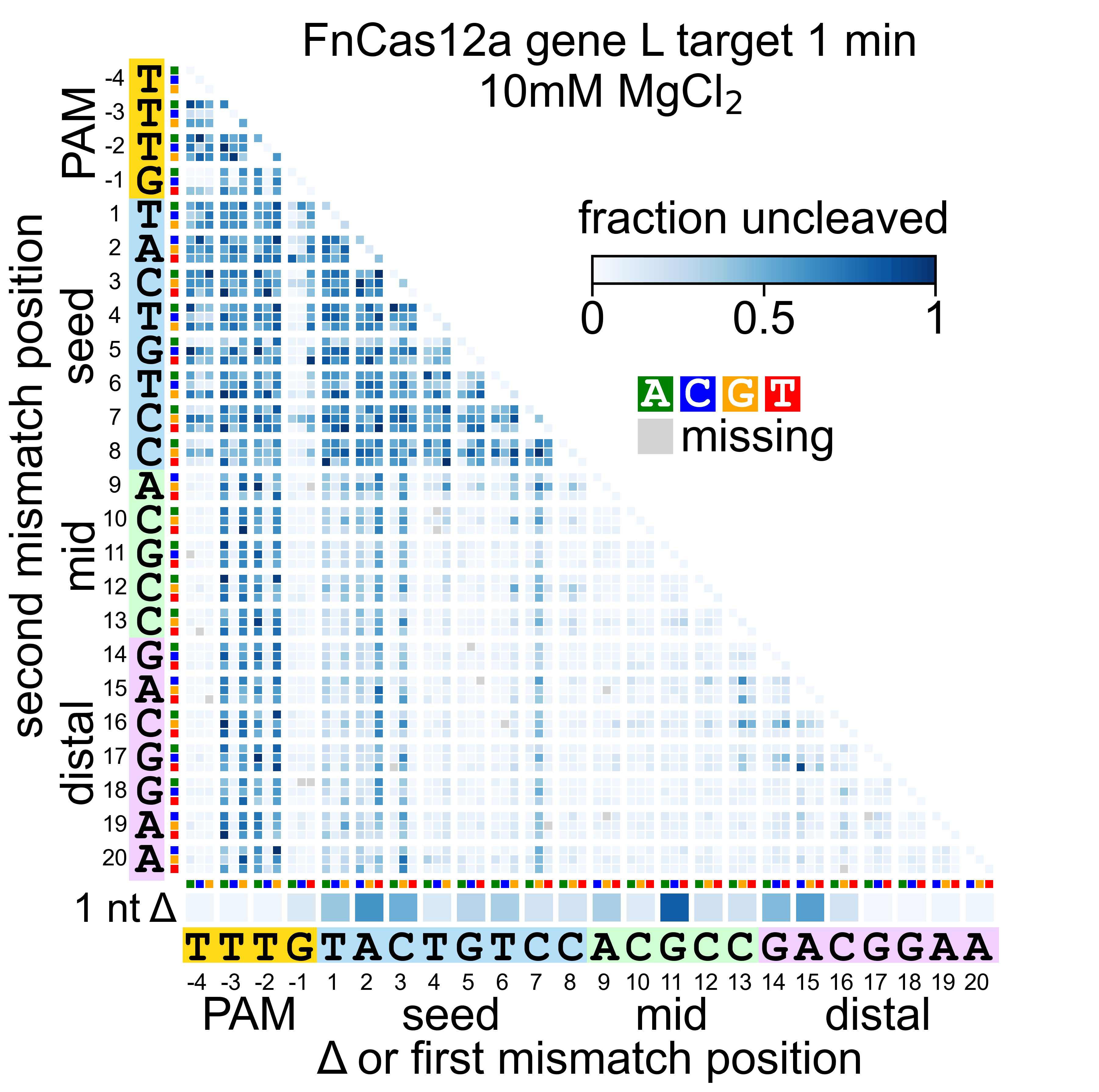

Supplement: Supplement 1 [file media-1.zip › Supplementary_Data_1/fraction_uncleaved_gifs/Fn_L_1_uncleaved.gif]

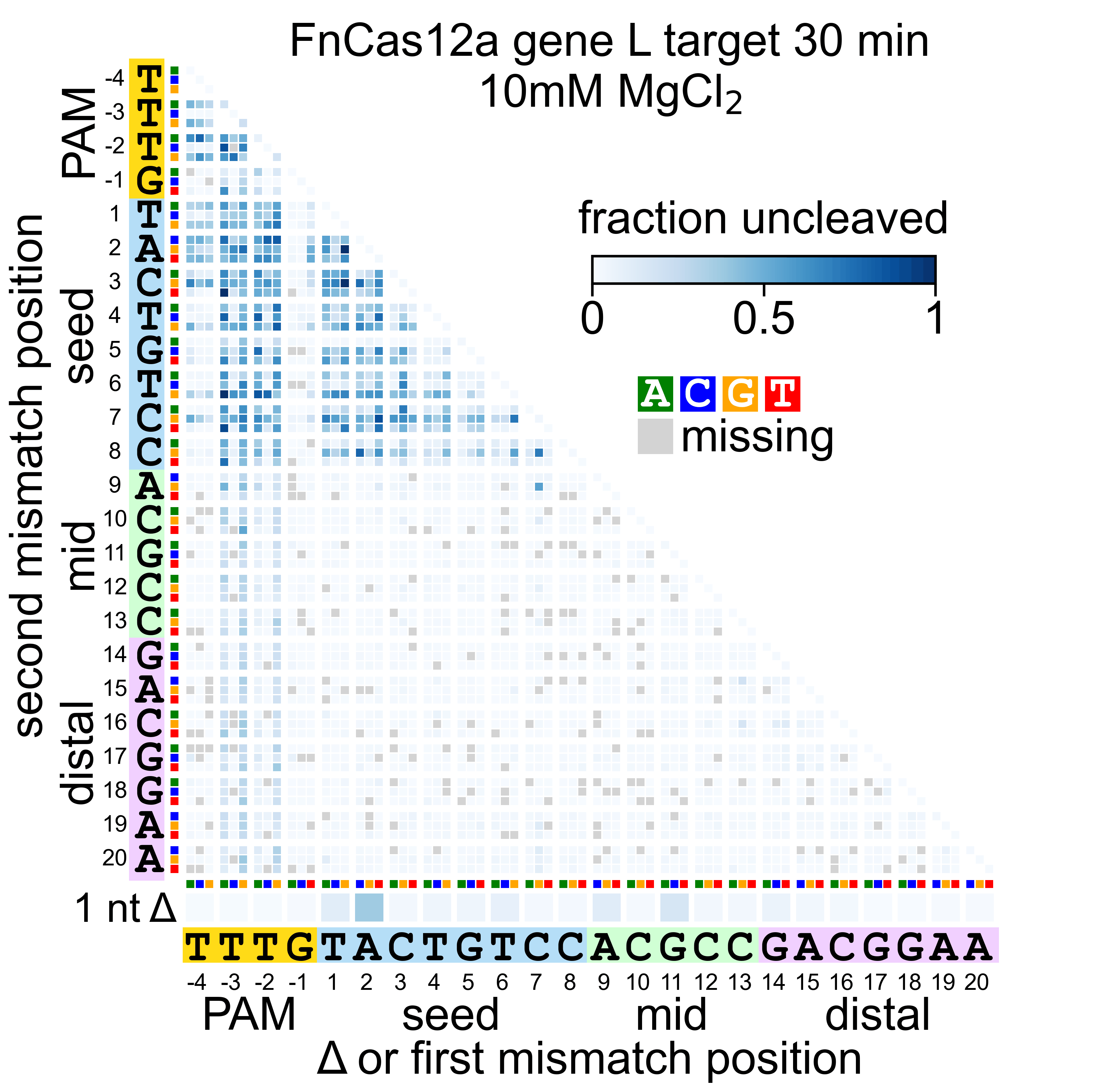

Supplement: Supplement 1 [file media-1.zip › Supplementary_Data_1/fraction_uncleaved_gifs/Fn_L_30_uncleaved.gif]

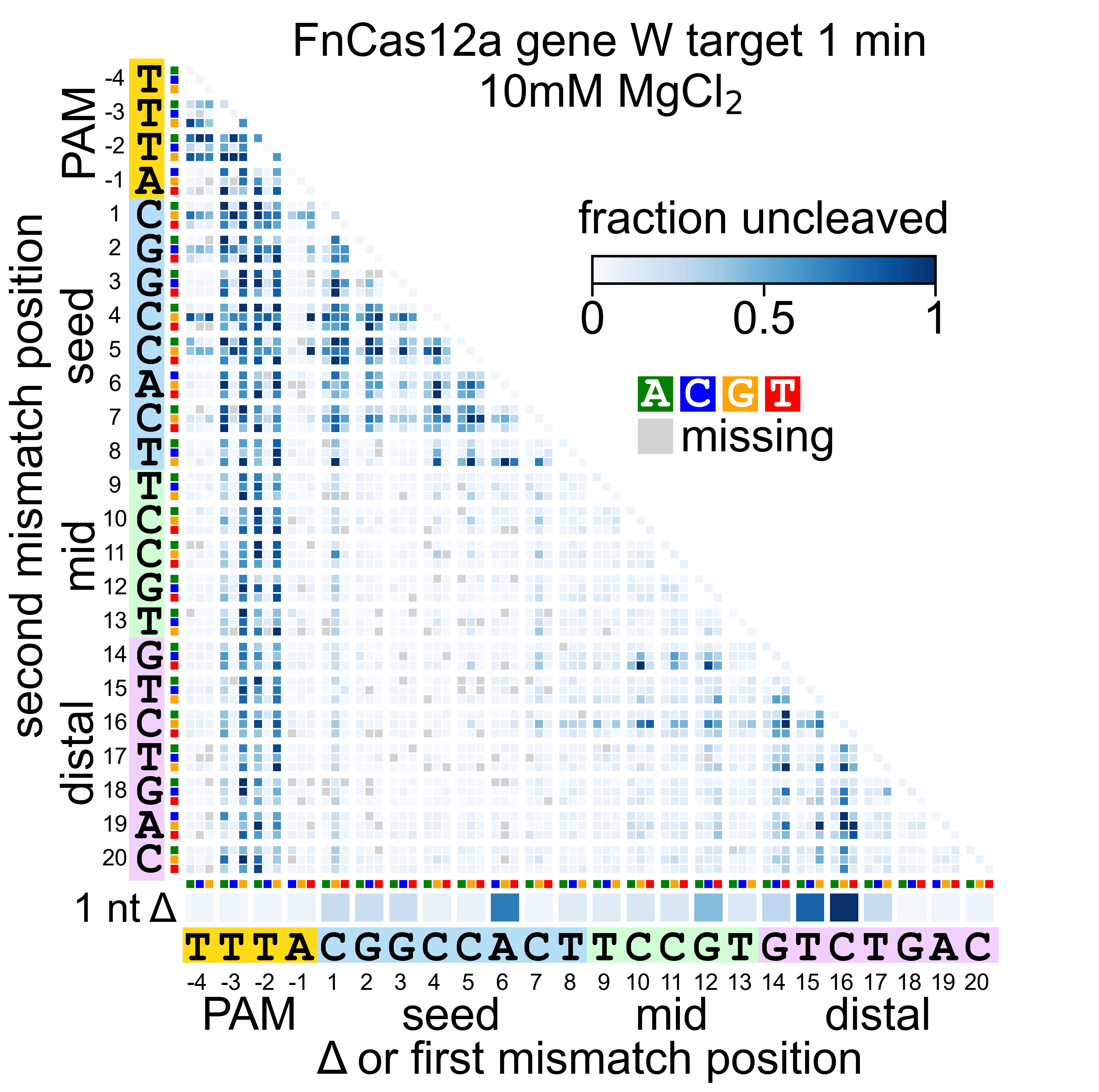

Supplement: Supplement 1 [file media-1.zip › Supplementary_Data_1/fraction_uncleaved_gifs/Fn_W_1_uncleaved.gif]

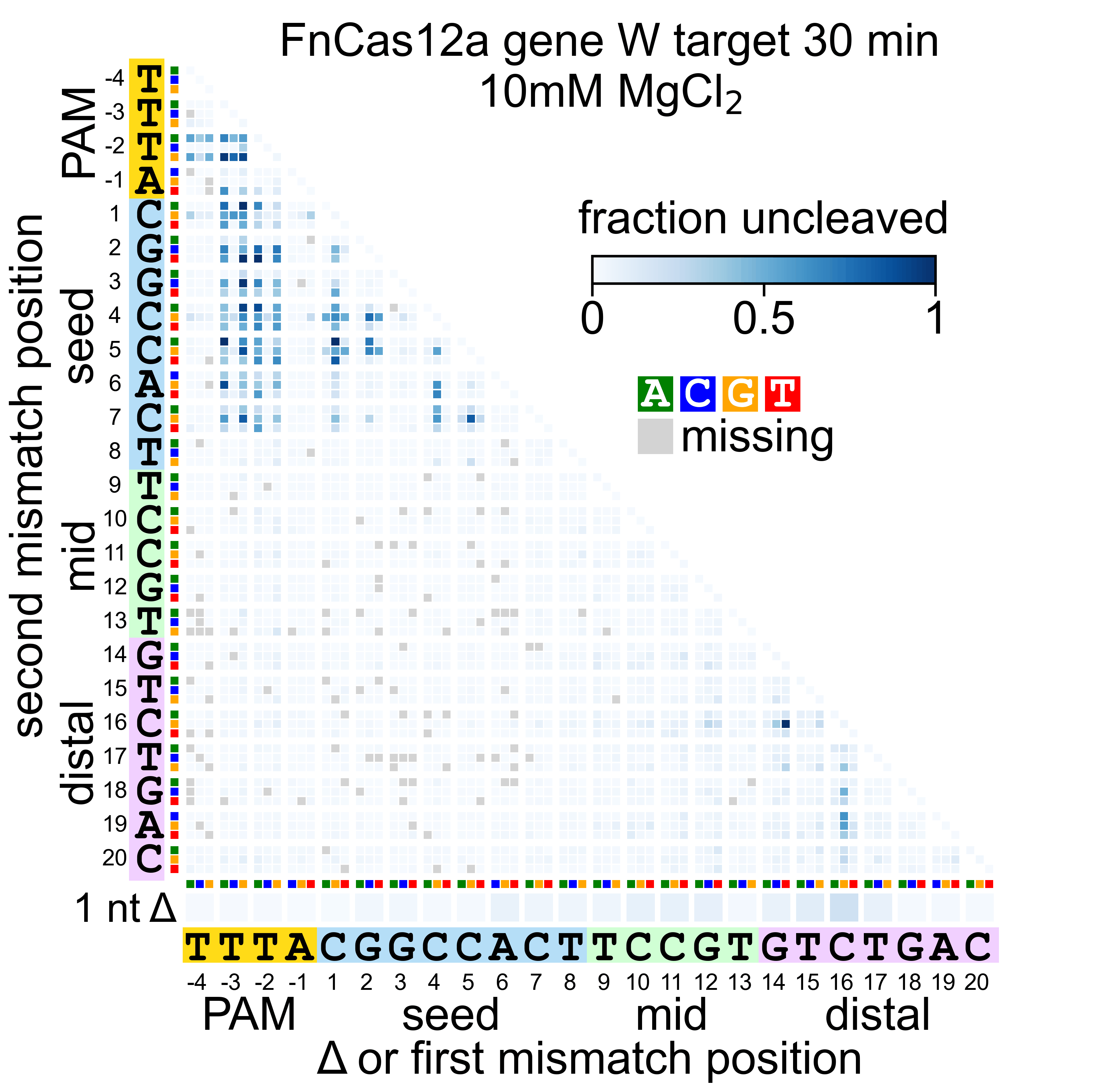

Supplement: Supplement 1 [file media-1.zip › Supplementary_Data_1/fraction_uncleaved_gifs/Fn_W_30_uncleaved.gif]

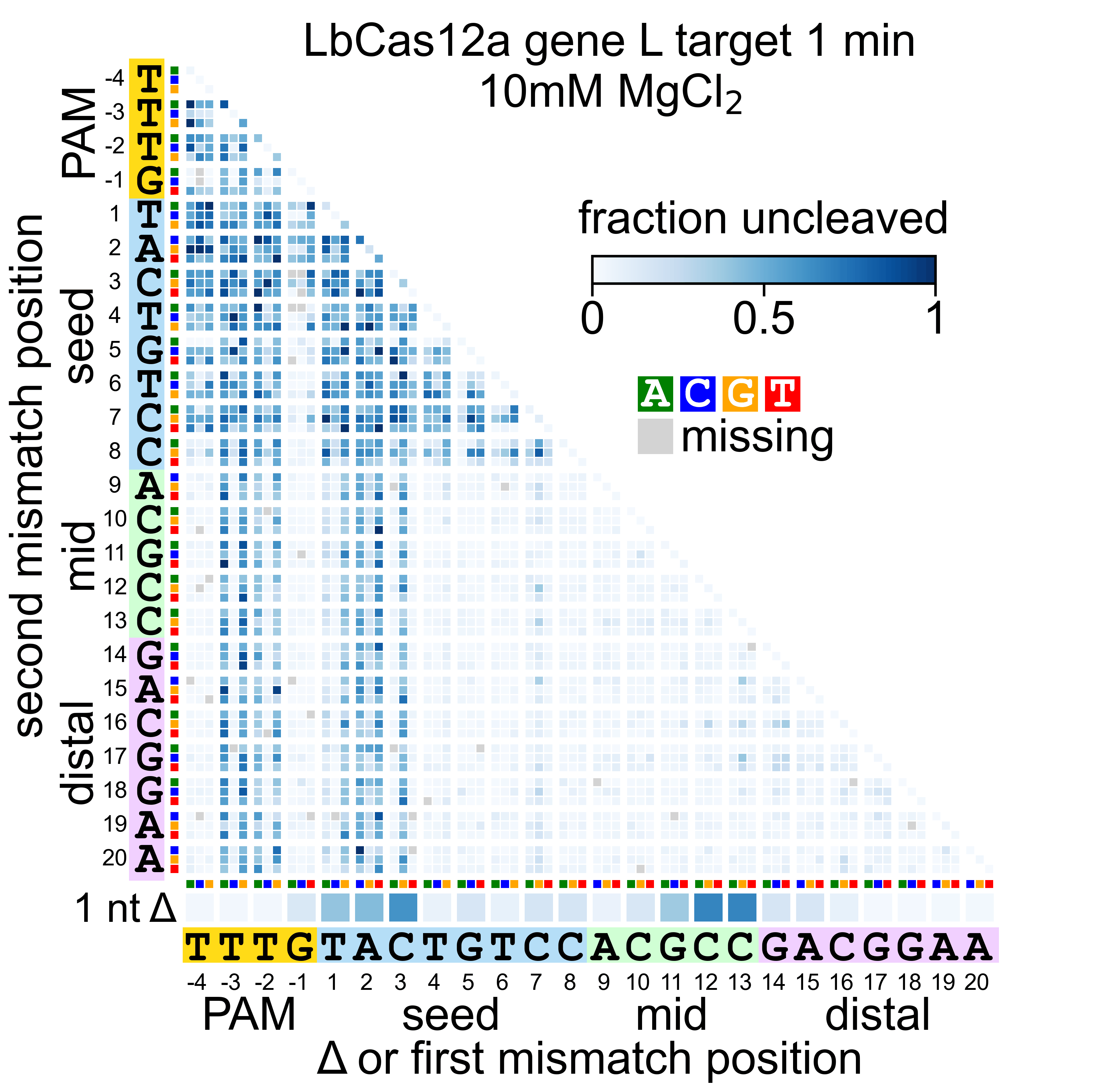

Supplement: Supplement 1 [file media-1.zip › Supplementary_Data_1/fraction_uncleaved_gifs/Lb_L_1_uncleaved.gif]

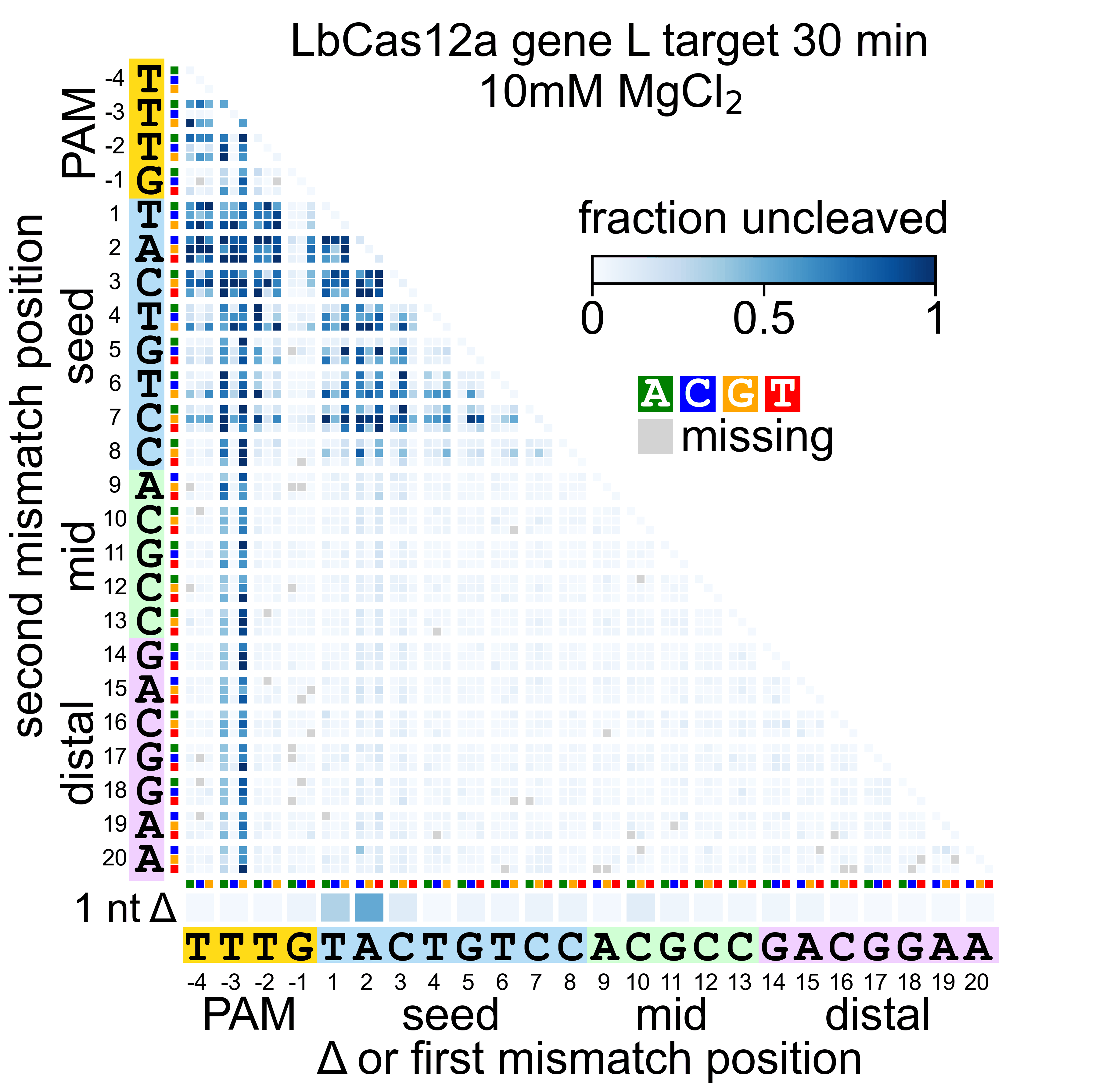

Supplement: Supplement 1 [file media-1.zip › Supplementary_Data_1/fraction_uncleaved_gifs/Lb_L_30_uncleaved.gif]

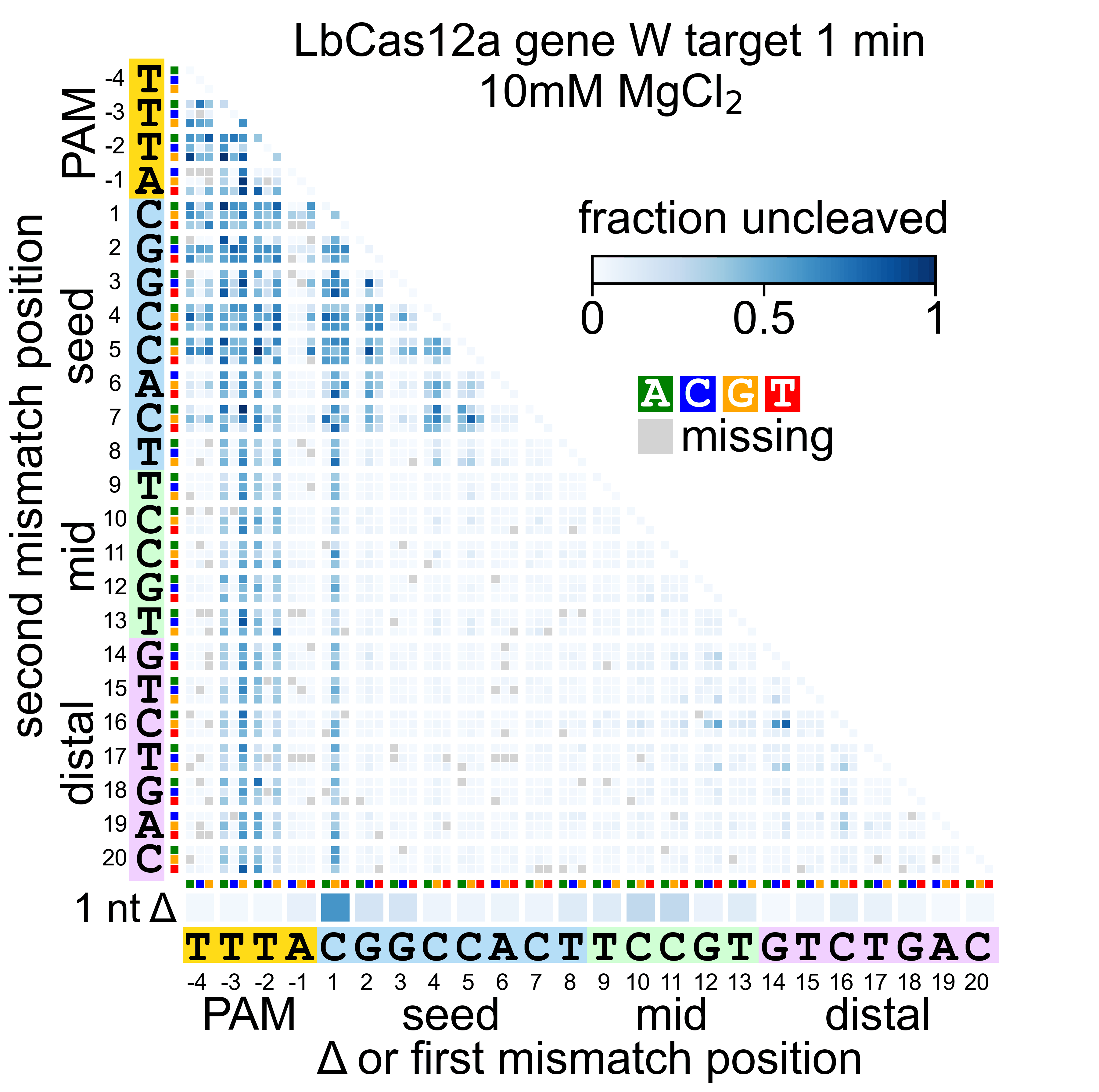

Supplement: Supplement 1 [file media-1.zip › Supplementary_Data_1/fraction_uncleaved_gifs/Lb_W_1_uncleaved.gif]

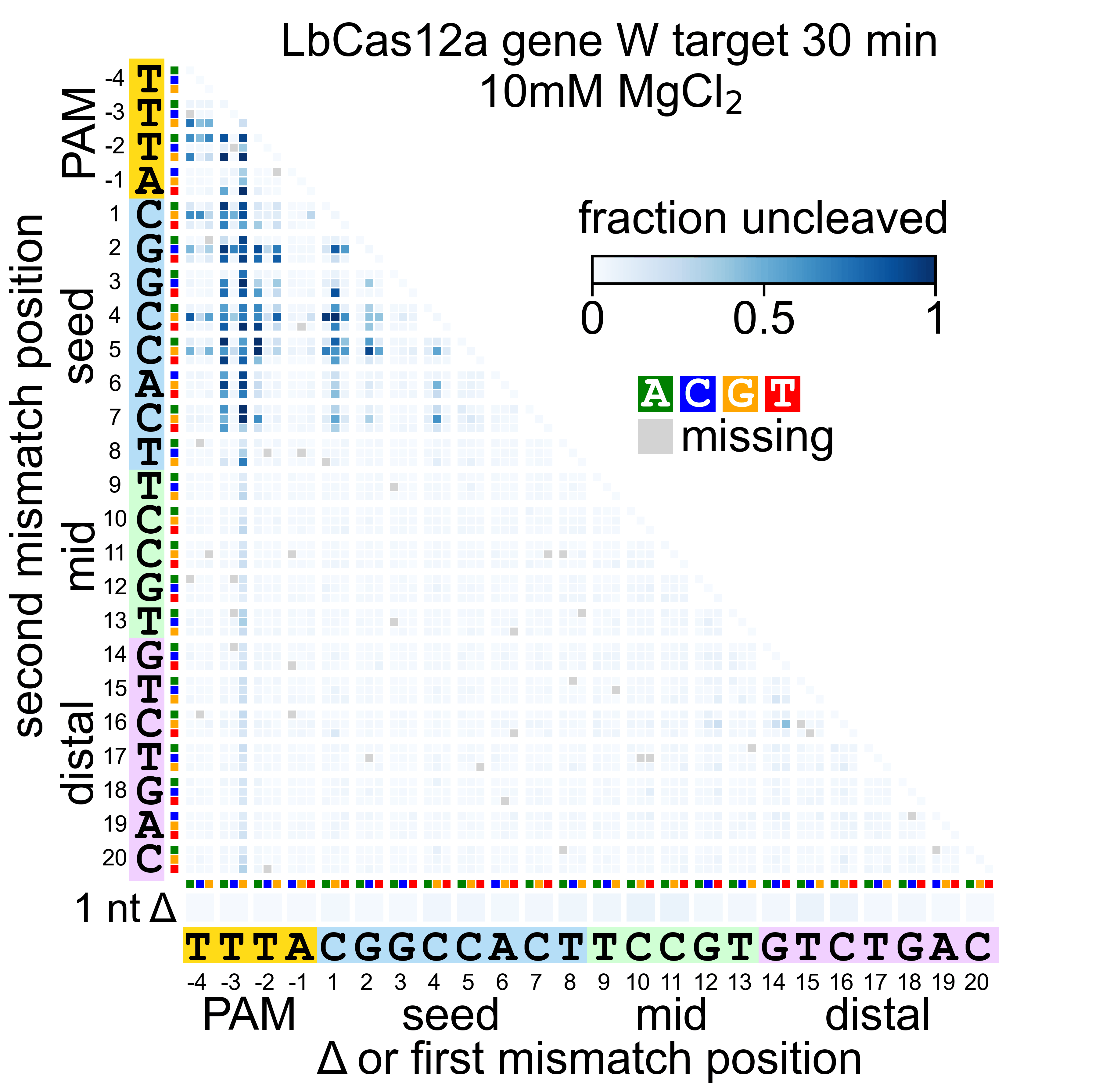

Supplement: Supplement 1 [file media-1.zip › Supplementary_Data_1/fraction_uncleaved_gifs/Lb_W_30_uncleaved.gif]
