# Supplementary material for: CRISPR-Cas12a exhibits metal-dependent specificity switching
Source: bioRxiv. 2023 Nov 29:2023.11.29.569287. Preprint. [Version 1] doi: 10.1101/2023.11.29.569287 (PMC10705449; doi:10.1101/2023.11.29.569287)
Supplement: Supplementary file 1 [file NIHPP2023.11.29.569287V1-supplement-1.pdf]

bioRxiv preprint doi: https://doi.org/10.1101/2023.11.29.569287; this version posted November 29, 2023. The copyright holder for this preprint (which was not certified by peer review) is the author/funder, who has granted bioRxiv a license to display the preprint in perpetuity. It is made available under aCC-BY 4.0 International license.

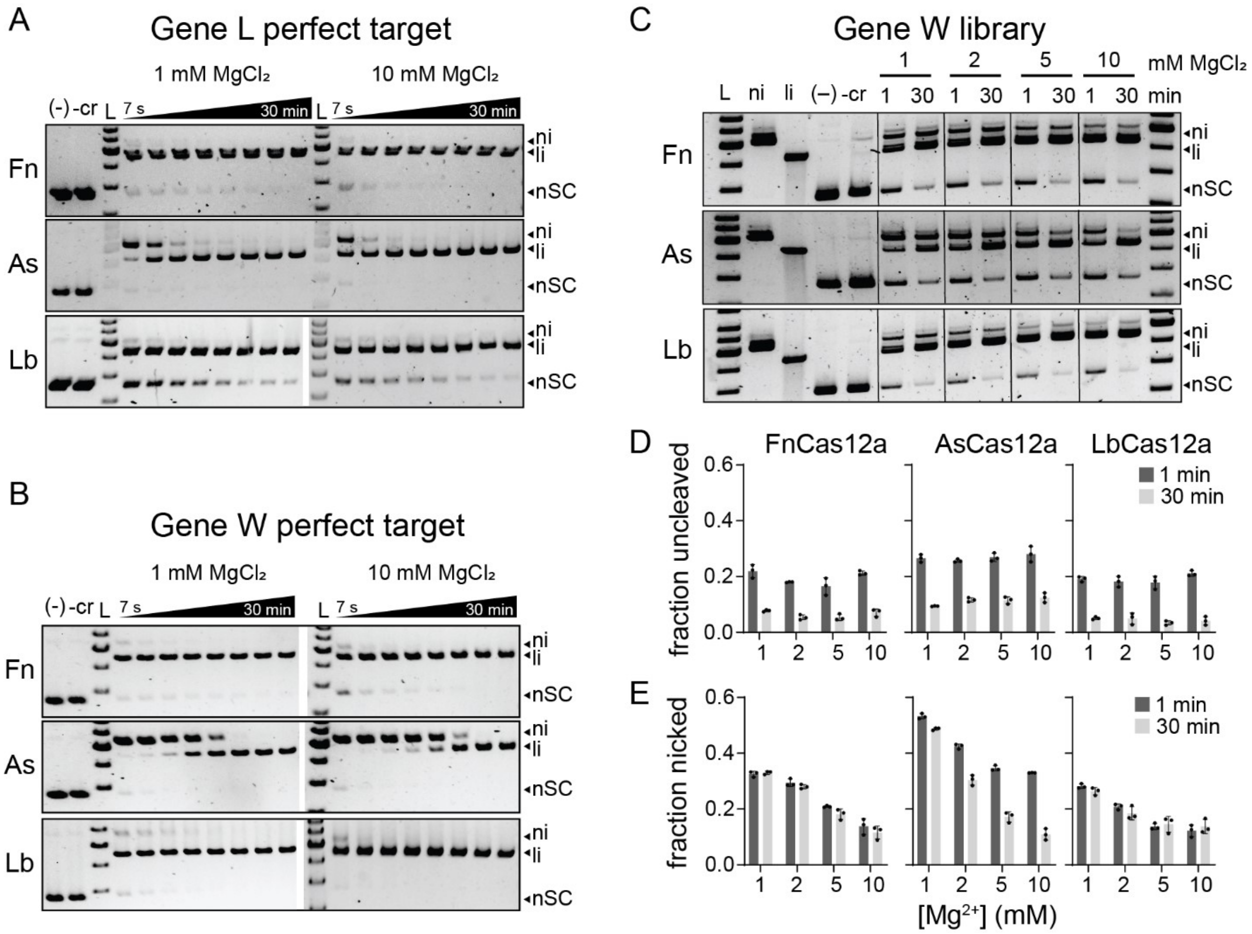

Figure S1: Cleavage of gene L and W perfect targets and gene W target library (Related to Figure 1)

A-B) Cleavage of the perfectly matched gene L (A) and gene W (B) targets by each Cas12a ortholog at 1 and 10 mM $MgCl_2$. The time points are 7 s, 15 s, 30 s, 1 min, 2 min, 5 min, 15 min, and 30 min. ni = nicked, li = linear, nSC = negatively supercoiled. (–) lane contains no protein, -cr contains protein but no crRNA. Both controls contained 10 mM $MgCl_2$ and were incubated at 37 °C for 30 min. Gel is representative of three replicates.

C) Gene W plasmid library cleavage at four Mg^{2+} concentrations for FnCas12a (Fn), AsCas12a (As), and LbCas12a (Lb). ni = nicked, li = linear, nSC = negatively supercoiled. (–) lane contains no protein, -cr contains protein but no crRNA. Both controls contained 10 mM $MgCl_2$ and were incubated at 37 °C for 30 min. Gel is representative of three replicates.

bioRxiv preprint doi: https://doi.org/10.1101/2023.11.29.569287; this version posted November 29, 2023. The copyright holder for this preprint (which was not certified by peer review) is the author/funder, who has granted bioRxiv a license to display the preprint in perpetuity. It is made available under aCC-BY 4.0 International license.

D-E) Quantification of fraction uncleaved (D) or nicked (E) for the gene W library. The average of three replicates is plotted, with individual data points shown as dots and error bars representing standard deviation.

bioRxiv preprint doi: https://doi.org/10.1101/2023.11.29.569287; this version posted November 29, 2023. The copyright holder for this preprint (which was not certified by peer review) is the author/funder, who has granted bioRxiv a license to display the preprint in perpetuity. It is made available under aCC-BY 4.0 International license.

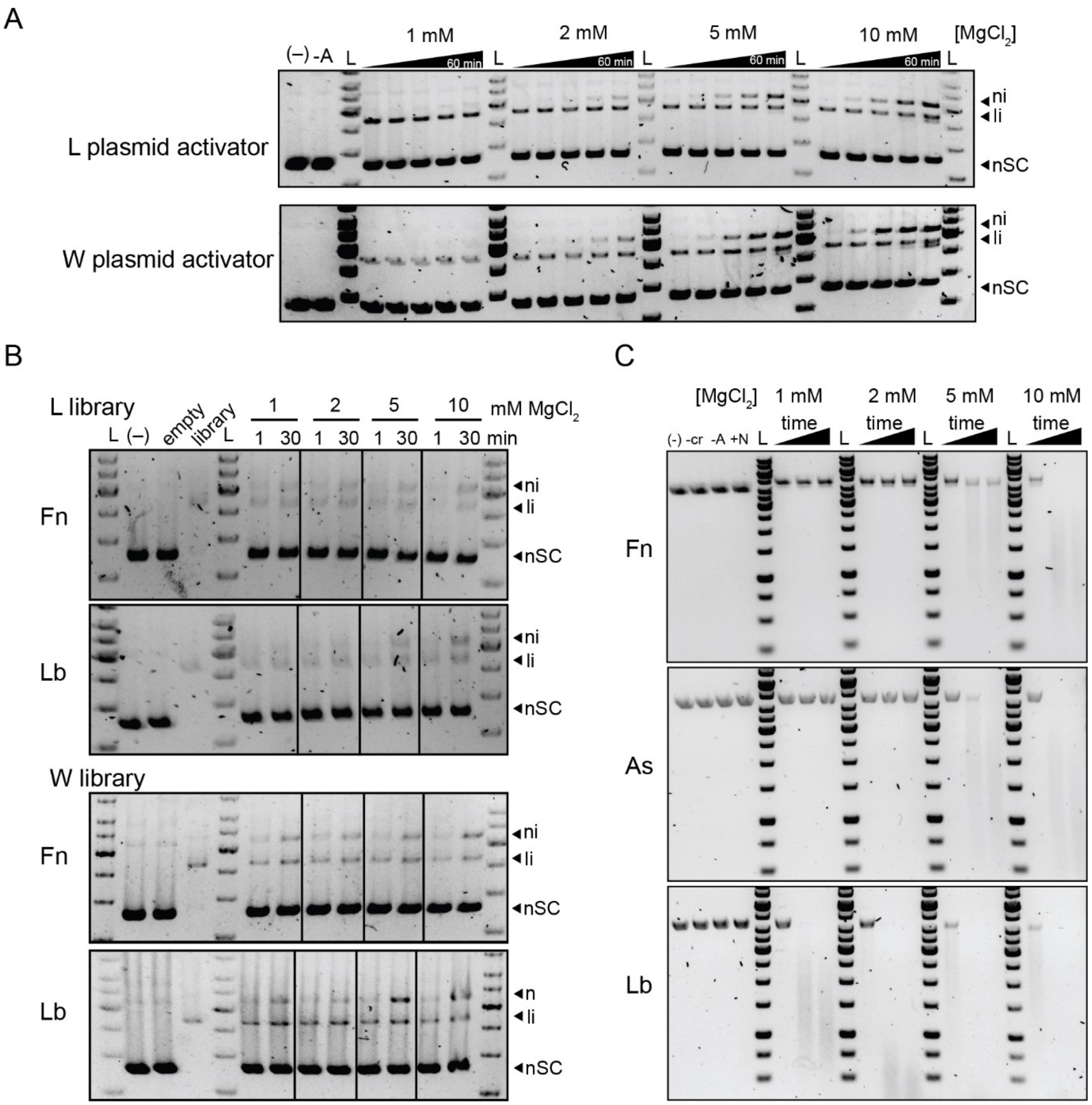

Figure S2: Collateral cleavage by Cas12a is Mg^{2+}-dependent (Related to Figure 1)

A) Cleavage of an empty pUC19 plasmid that lacks any complementarity to the crRNA by LbCas12a activated with either a gene L and gene W target pUC19 plasmid. The empty plasmid was in 10X excess of the activator target plasmid. The target plasmid was linearized by the first time point, and additional cleavage products observed at subsequent time points are most likely due to cleavage of the empty pUC19 plasmid. Time points: 1 min, 5 min, 15 min, 30 min, 60 min. The first two lanes are controls with empty pUC19 plasmid without any protein added (–)

bioRxiv preprint doi: https://doi.org/10.1101/2023.11.29.569287; this version posted November 29, 2023. The copyright holder for this preprint (which was not certified by peer review) is the author/funder, who has granted bioRxiv a license to display the preprint in perpetuity. It is made available under aCC-BY 4.0 International license.

or with Cas12a-crRNA but without the activator target plasmid added (-A) at 10 mM $MgCl_2$ incubated at 37 °C for the longest time point.

B) Cleavage of an empty pUC19 plasmid that lacks any complementarity to the crRNA by Fn or LbCas12a activated with either the gene L and gene W target plasmid library. The empty plasmid was in 10X excess of the plasmid library. The first three lanes are controls with empty pUC19 plasmid without any protein added (–) or with Cas12a-crRNA but with only the empty pUC19 plasmid (empty) or the plasmid library (library) at 10 mM $MgCl_2$ incubated at 37 °C for the longest time point.

C) Cleavage of single-stranded M13 phage DNA by Cas12a activated with a short double-stranded DNA activator. The time points were 15 s, 30 min, and 60 min. The first four lanes are controls with M13 DNA without any protein added (–), M13 DNA with only Cas12a added (-cr), Cas12a-crRNA but without the activator DNA added (-A), or Cas12a-crRNA with a non-targeting dsDNA oligo added (+N). All controls contained 10 mM $MgCl_2$ and were incubated at 37 °C for the longest time point.

bioRxiv preprint doi: https://doi.org/10.1101/2023.11.29.569287; this version posted November 29, 2023. The copyright holder for this preprint (which was not certified by peer review) is the author/funder, who has granted bioRxiv a license to display the preprint in perpetuity. It is made available under aCC-BY 4.0 International license.

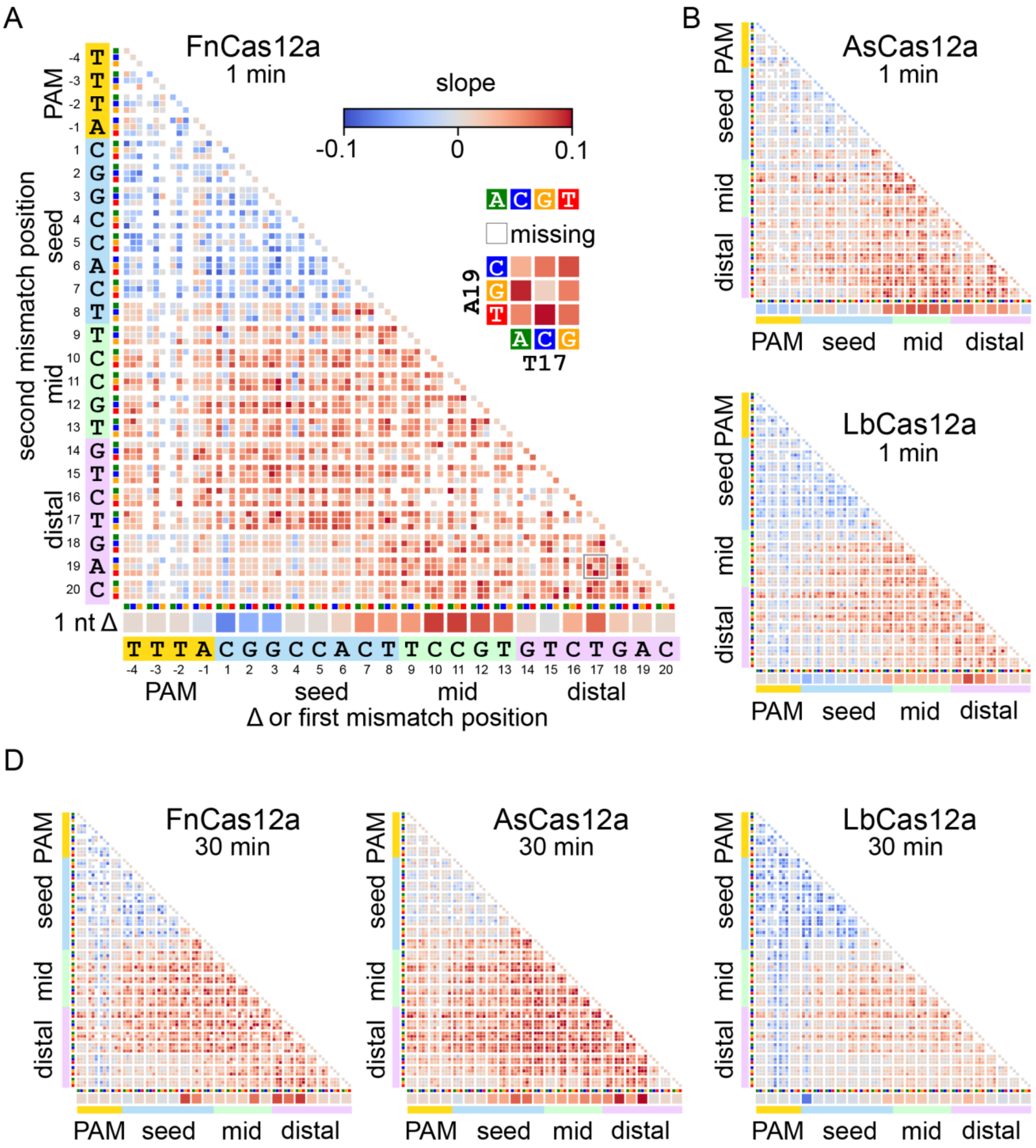

Figure S3: Slope heatmaps for the gene W target (Related to Figure 3)

A) Heatmap plotting slopes for fully cleaved DNA versus Mg^{2+} as determined in Figure 3A-B. The heatmap is for the gene W target plasmid library following 1 min cleavage by FnCas12a. Missing sequences are represented by white boxes.

bioRxiv preprint doi: https://doi.org/10.1101/2023.11.29.569287; this version posted November 29, 2023. The copyright holder for this preprint (which was not certified by peer review) is the author/funder, who has granted bioRxiv a license to display the preprint in perpetuity. It is made available under aCC-BY 4.0 International license.

B) Slope heatmaps of the gene W target plasmid library following 1 min cleavage by AsCas12a or LbCas12a.

C) Slope heatmaps of the gene W target plasmid library following 30 min cleavage by all three orthologs.

bioRxiv preprint doi: https://doi.org/10.1101/2023.11.29.569287; this version posted November 29, 2023. The copyright holder for this preprint (which was not certified by peer review) is the author/funder, who has granted bioRxiv a license to display the preprint in perpetuity. It is made available under aCC-BY 4.0 International license.

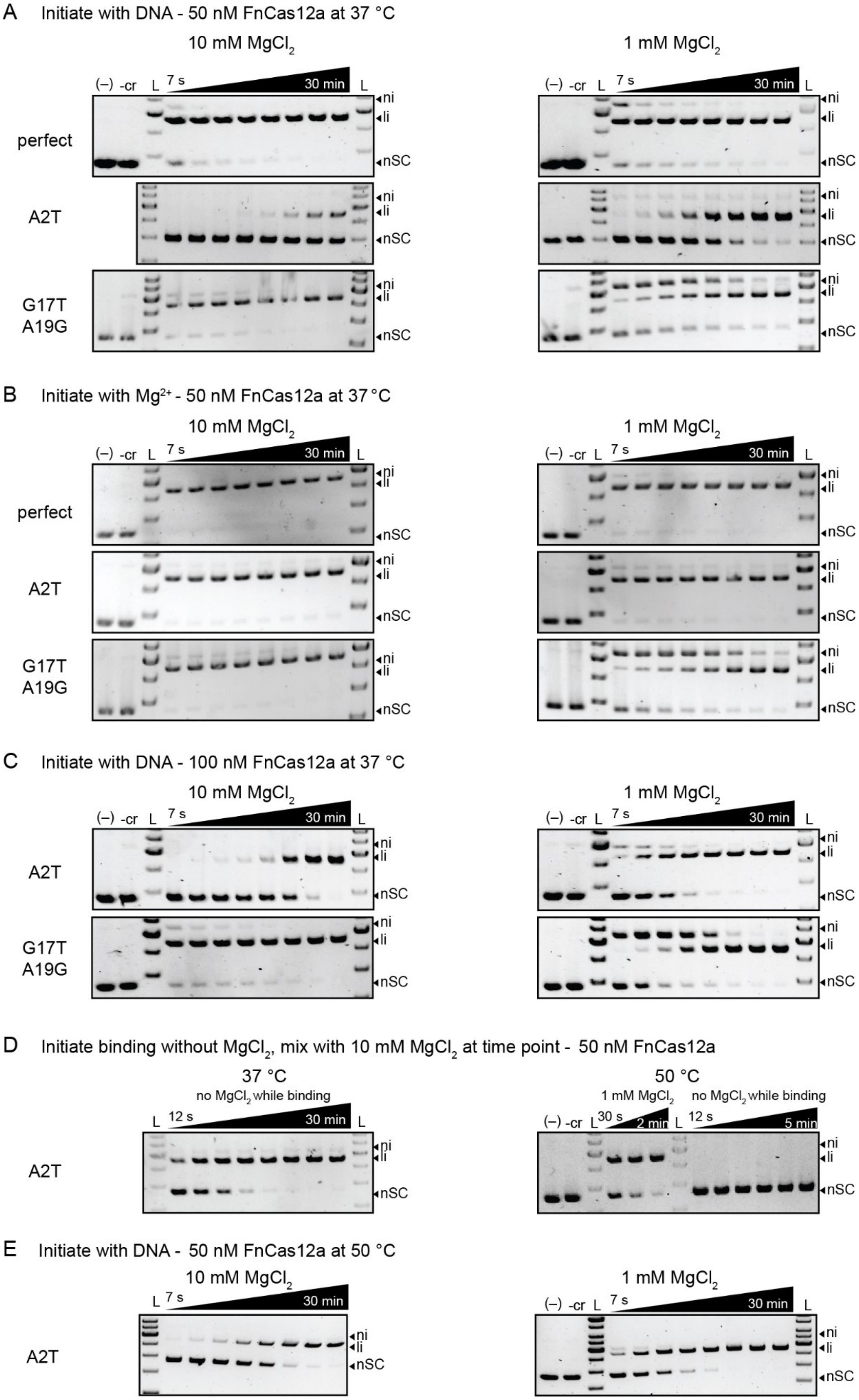

bioRxiv preprint doi: https://doi.org/10.1101/2023.11.29.569287; this version posted November 29, 2023. The copyright holder for this preprint (which was not certified by peer review) is the author/funder, who has granted bioRxiv a license to display the preprint in perpetuity. It is made available under aCC-BY 4.0 International license.

Figure S4: Representative agarose gels for cleavage assays quantified in Figure 4 (Related to Figure 4)

Cleavage of plasmids bearing the indicated gene L target sequence by FnCas12a for the conditions described below. The gene L sequence either perfectly matched the crRNA (perfect) or contained a seed (A2T) or two PAM-distal (G17T A19G) mutations. For all gels, (–) indicates a control in which no protein was added to the DNA and -cr indicates a control in which only Cas12a without crRNA was added to DNA before incubating at 37 °C for 30 min. Controls were performed at the same $MgCl_2$ concentration as the cleavage reaction. The time points for all gels were 7 s, 15 s, 30 s, 1 min, 2 min, 5 min, 15 min and 30 min, unless otherwise indicated. Each gel is a representative of at least three replicates. ni = nicked, li = linear, nSC = negatively supercoiled.

A) Gels associated with the quantified data shown in Figure 4B and 4D (50 nM RNP) and 4E (37 °C) in which Cas12a cleavage was initiated by mixing Cas12a-crRNA RNP together with DNA in the presence of the indicated concentration of $MgCl_2$.

B) Gels associated with the quantified data shown in Figure 4C in which Cas12a-crRNA RNP was first incubated with DNA for 30 min prior to addition of the indicated concentration of $MgCl_2$ to initiate cleavage.

C) Gels associated with the quantified data shown in Figure 4D (100 nM RNP) in which Cas12a cleavage was initiated by mixing Cas12a-crRNA RNP together with DNA in the presence of the indicated concentration of $MgCl_2$.

D) Gels associated with the quantified data shown in Figure 4E (37 °C) in which binding was initiated in the absence of $MgCl_2$. For the gel on the left, RNP-DNA binding reaction aliquots from the binding reaction were mixed with 10 mM $MgCl_2$ for 5 s prior to quenching. For the gel on the right, we did not observe any cleavage when the same type of experiment was performed at 50 °C (last 6 lanes), although Cas12a retains cleavage activity in the presence of 1 mM $MgCl_2$. This may be due to instability of the protein at 50 °C in the absence of divalent metal ions.

E) Gels associated with the quantified data shown in Figure 4E (50 °C) in which Cas12a cleavage was initiated by mixing Cas12a-crRNA RNP together with DNA in the presence of the indicated concentration of $MgCl_2$.

bioRxiv preprint doi: https://doi.org/10.1101/2023.11.29.569287; this version posted November 29, 2023. The copyright holder for this preprint (which was not certified by peer review) is the author/funder, who has granted bioRxiv a license to display the preprint in perpetuity. It is made available under aCC-BY 4.0 International license.

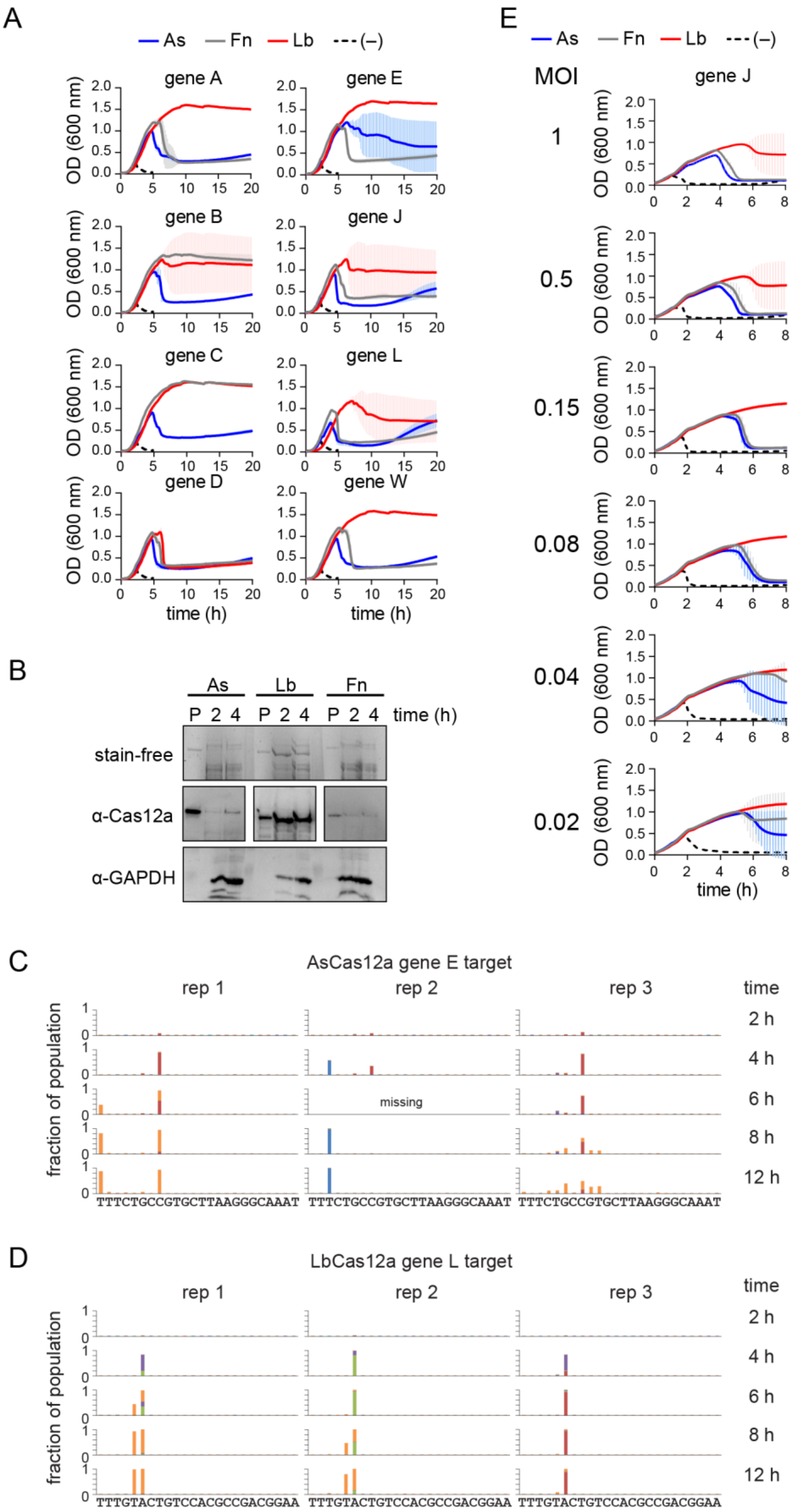

bioRxiv preprint doi: https://doi.org/10.1101/2023.11.29.569287; this version posted November 29, 2023. The copyright holder for this preprint (which was not certified by peer review) is the author/funder, who has granted bioRxiv a license to display the preprint in perpetuity. It is made available under aCC-BY 4.0 International license.

Figure S5: Growth curves, Western blots and individual replicates for phage escape experiments (Related to Figure 5)

A) Growth curves (optical density at 600 nm wavelength vs. time) for *E. coli* cultures expressing each Cas12a ortholog and a crRNA targeting the indicated gene. Cultures were infected with phage λ_{vir} at an OD_{600} of 0.3. The average of three replicates are shown and error bars represent standard deviation. Large error bars indicate that some replicates did not undergo lysis while some did.

B) Western blot analysis of Cas12a ortholog expression in *E. coli* cultures used for phage escape assays. The top panel shows the SDS-PAGE gel visualized using 2,2,2-trichloroethanol stain-free imaging. Purified AsCas12a, FnCas12a or LbCas12a (~150 ng) was loaded in the first of three lanes, followed by lysates of cultures expressing each ortholog harvested at the indicated time points. The middle panel shows the Western blots using antibodies against each individual Cas12a ortholog. The bottom panel shows a Western blot using an antibody against *E. coli* GAPDH as a loading control for the lysates.

C-D) Bar graphs showing double mutations that arose over time for three replicates of cultures expressing AsCas12a bearing a crRNA targeting gene E (C) or LbCas12a bearing a crRNA targeting gene L. A second mutation arose after an initial mutation for two of three replicates for each set of cultures.

E) Growth curves for *E. coli* cultures expressing each Cas12a ortholog and a crRNA targeting gene J infected with the indicated MOI of phage λ_{vir} at an OD_{600} of 0.3. The average of three replicates are shown and error bars represent standard deviation. Large error bars indicate that some replicates did not undergo lysis while some did.

Supplementary Data 1: Animated gifs showing the abundance of each mutant sequence in the uncleaved or nicked fraction. Each gif shows all four Mg^{2+} concentrations for a given ortholog and gene at a given time point.

Supplementary Data 2: All plasmids, primers and oligonucleotides used in this study.
